# Obesity, hypertension, diabetes mellitus, and hypercholesterolemia in Korean adults before and during the COVID-19 pandemic: a special report of the 2020 Korea National Health and Nutrition Examination Survey

**DOI:** 10.4178/epih.e2022041

**Published:** 2022-04-25

**Authors:** Ga Bin Lee, Yoonjung Kim, Suyeon Park, Hyeon Chang Kim, Kyungwon Oh

**Affiliations:** 1 Departments of Preventive Medicine, Yonsei University College of Medicine, Seoul, Korea; 2 Division of Health and Nutrition Survey and Analysis, Bureau of Chronic Disease Prevention and Control, Korea Disease Control and Prevention Agency, Cheongju, Korea

**Keywords:** Chronic disease, COVID-19, Pandemics, Prevalence, Korea National Health and Nutrition Examination Survey

## Abstract

**OBJECTIVES:**

We investigated trends in obesity, hypertension, diabetes, and hypercholesterolemia before and during the coronavirus disease 2019 (COVID-19) pandemic in the Korean adult population.

**METHODS:**

Data from 60,098 participants in the Korea National Health and Nutritional Examination Survey between 2011 and 2020 aged ≥19 were used. The age-standardized prevalence and annual percent changes (APCs) were calculated for obesity (body mass index ≥25 kg/m^2^), hypertension (systolic/diastolic blood pressure ≥140/90 mmHg or under treatment), diabetes (hemoglobin A1c ≥6.5%, fasting glucose ≥126 mg/dL, physician diagnosis, or under treatment), and hypercholesterolemia (total cholesterol ≥240 mg/dL or under treatment).

**RESULTS:**

Over the past decade (2011-2020), the age-standardized APCs (95% confidence intervals) for obesity, hypertension, diabetes and hypercholesterolemia were 3.0% (2.1 to 3.8), 0.1% (-1.3 to 1.5), 1.5% (-1.0 to 4.0) and 8.0% (5.7 to 10.3), respectively, in men; and -0.2% (-1.5 to 1.2), -0.5% (-1.9 to 0.9), -0.1% (-2.3 to 2.2) and 5.9% (3.9 to 8.0), respectively, in women. In 2020 compared to the previous 3 years (2017-2019), obesity, hypertension, diabetes, and hypercholesterolemia increased in men (6.0, 1.8, 1.9, and 2.8%p, respectively), but an increase was not apparent in women (2.5, -1.1, 0.8, and 0.7%p, respectively).

**CONCLUSIONS:**

An increase in major chronic diseases was observed in Korean adults, especially men, during the COVID-19 pandemic. In order to reduce the burden of cardiovascular and metabolic diseases in the future, effective intervention strategies need to be developed according to the characteristics of the target groups.

## GRAPHICAL ABSTRACT

[Fig f2-epih-44-e2022041]

## INTRODUCTION

Non-communicable diseases (NCDs) account for more than half of the entire disease burden according to the 2019 Global Burden of Cardiovascular Disease study, an ongoing multinational collaboration of 204 countries [1]. In particular, from 1990 to 2019, the prevalent cases of cardiovascular disease (CVD) nearly doubled and the number of CVD deaths steadily increased worldwide [2]. Accordingly, the burden of CVD, as assessed by disability-adjusted life years and years of life lost, steadily rose over that period. Well-known modifiable CVD risk factors, such as obesity and elevated levels of glucose/lipids, are also steadily increasing [2,3]. Although the prevalence of high blood pressure significantly decreased in the high-income Asia-Pacific region between 1975 and 2015 [4], the highest absolute burden of hypertension was observed in that region [5].

The prevalence of chronic diseases in 2020 is worth reporting from the standpoint of public health. The sudden outbreak of coronavirus disease 2019 (COVID-19) changed our society in many ways. People have been required to maintain physical distancing in the public and private sectors [6]. However, unintended results have been reported. Previous studies have reported deteriorations in health behavior during the COVID-19 pandemic, such as increased sedentary behaviors and unhealthy eating habits [7-9]. Given that the impact of deleterious health behaviors on cardio-metabolic health is well-described [10], the results of social changes in 2020 on health outcomes need to be reported. Moreover, no previous studies have used nationally representative data to analyze the prevalence of chronic diseases after and during the COVID-19 pandemic.

Therefore, this study analyzed data from the Korea National Health and Nutritional Examination Survey (KNHANES) to examine the trends in the prevalence of obesity, hypertension, diabetes mellitus, and hypercholesterolemia over a 10-year period, with the specific aim of observing whether there were significant changes in the prevalence of chronic diseases in 2020.

## MATERIALS AND METHODS

### Study population

The current study utilized data obtained from the KNHANES between 2011 and 2020. The KNHANES is an ongoing national surveillance system in Korea that has been assessing the population’s health and nutritional status since 1998. The KNHANES consists of 3 component surveys: a health interview, a health examination, and a nutrition survey. The survey has been conducted cross-sectionally by the Korea Disease Control and Prevention Agency (KDCA) in each year. Detailed information on the aims and design of the KNHANES has been previously reported [11-13]. Briefly, the KNHANES is a multistage probability sample of the civilian non-institutionalized Korean population. Despite of the COVID-19 pandemic in 2020, NHANES was conducted and strictly followed precaution for COVID-19 including wearing N94 mask, sanitizing hands every examination, checking body temperature, and excluding household with member who have similar symptom or confirmed for COVID-19. The participation rate in 2020 is 74.0%, which is similar to that of 74.7% in 2019. Each year, the survey yields a new sample of about 10,000 individuals aged 1 year or over. Data from KNHNAES participants ≥ 19 years old were included in the present analysis (n= 60,098).

### Measurements

In the KNHANES, certified field staff performed health interviews and health examinations using a standardized procedure at mobile examination centers. All participants provided informed consent. Blood samples were collected after participants had fasted for at least 8 hours and were analyzed in a certified laboratory on the same day. Height was measured using wall-mounted stadiometers (SECA 225 [2011 to June 2019], SECA 274 [July 2019 to 2020]; Seca GmbH, Hamburg, Germany), and weight was measured using a portable electronic scale. Body mass index (BMI) was calculated as weight in kilograms divided by the square of height in meters. Obesity and severe obesity were defined as a BMI ≥ 25 kg/m^2^ and a BMI ≥ 30 kg/m^2^, respectively [14]. Blood pressure was measured 3 times on the right arm of the subject in a sitting position after 5 minutes of rest using a mercury sphygmomanometer in 2011-2019 (Baumanometer; Baum, Copiague, NY, USA) or a non-mercurial sphygmomanometer in 2020 (Greenlight 300; Accoson, Ayrshire, UK). Prevalent hypertension was determined if the participants had a systolic blood pressure ≥ 140 mmHg or a diastolic blood pressure ≥ 90 mmHg, or was. currently taking antihypertensive medications [15]. Prevalent diabetes was determined if the participants had hemoglobin A1c ≥ 6.5%, a fasting glucose level ≥ 126 mg/dL, or a self-reported diagnosis by a physician, or was currently taking hypoglycemic medications or insulin [16]. Prevalent hypercholesterolemia was determined if the participants had fasting total cholesterol levels ≥ 240 mg/dL or were currently taking lipid-lowering drugs [17]. Household income was calculated as the monthly household income divided by the square root of the number of persons in the household, and was categorized into quintiles according to age and gender.

### Statistical analysis

All statistical analyses were performed using SAS version 9.4 (SAS Institute Inc., Cary, NC, USA) and Joinpoint Regression Program version 4.1.1.1 (US National Cancer Institute, Bethesda, MD, USA). To achieve representativeness of the Korean population, the sampling weights assigned to participants were applied in all analyses. The prevalence of obesity, hypertension, diabetes, and hypercholesterolemia according to gneder and household income level were age-standardized based on the 2005 Korean population projections using SAS (PROC SURVEYREG). The prevalence according to age was presented as crude rates. Differences in estimates and 95% confidence intervals between the periods were calculated using SAS (PROC SURVEYREG). The estimates and standard errors obtained from SAS were input into the Joinpoint program, and the annual percent change (APC) was calculated.

### Ethics statement

All procedures and protocols of the study were approved by the Institutional Review Board of the KDCA (2011-14, 2018). From 2015 to 2017, ethical approval was waived by the Bioethics and Safety Act.

## RESULTS

Table 1 presents the general characteristics of the KNHANES participants from 2011 to 2020. Almost half of the participants consisted of men, and the mean age was the mid-40s. Participants were evenly distributed across all age groups. The mean BMI, systolic blood pressure, and fasting blood glucose steadily increased from 2011 to 2020.

**Figure 1.**
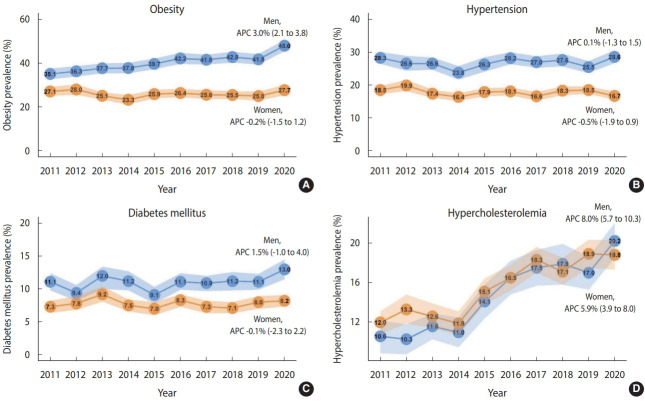
Trends in prevalence and annual percent change (APC) of obesity (A), hypertension (B), diabetes mellitus (C), and hypercholesterolemia (D). Numbers indicate the point estimates of the prevalence, and shaded areas indicate the 95% confidence interval. Age-standardized prevalence was calculated using the 2005 population projections for Korea. Values are presented as APC (95% CI).

Figure 1 depicts the trends in prevalence and APCs of obesity, hypertension, diabetes, and hypercholesterolemia. The annual prevalence and APCs from 2011 to 2020 of these chronic diseases were provided stratified by gender, age, and income level in Supplementary Materials 1-4. The prevalence of obesity steadily increased; however, the increase was largely limited to men (APC, 3.0%; 95% CI, 2.1 to 3.8), and there was little change in women (APC, -0.2%; 95% CI, -1.5 to 1.2). The prevalence of severe obesity (defined as BMI ≥ 30 kg/m^2^) increased significantly both in men (APC, 7.9%; 95% CI, 4.6 to 11.2) and in women (APC, 3.7%; 95% CI, 1.0 to 6.5) (Supplementary Material 5). There were no apparent changes in the prevalence of hypertension and diabetes; the APC (95% CI) of hypertension was 0.1% (-1.3 to 1.5) for men and -0.5% (-1.9 to 0.8) for women, and the APC (95% CI) of diabetes was 1.5% (-1.0 to 4.0) for men and -0.1% (-2.3 to 2.2) for women. Hypercholesterolemia increased rapidly in both men and women, with an APC (95% CI) of 8.0% (5.7 to 10.3) for men and 5.9% (3.9 to 8.0) for women.

Table 2 presents the changes in the prevalence of obesity, hypertension, diabetes, and hypercholesterolemia in 2020 compared to the mean of the last 3 years (2017-2019) before the COVID-19 pandemic. Compared to the average values from the previous 3 years (2017-2019), the prevalence of obesity, hypertension, diabetes, and hypercholesterolemia in 2020 increased in men, although the increase in hypertension was not statistically significant. The prevalence of these conditions increased by 6.0%p (95% CI, 3.2 to 8.8), 1.8%p (95% CI, -0.5 to 4.0), 1.9%p (95% CI, 0.3 to 3.5), and 2.8%p (95% CI, 0.8 to 4.9), respectively. The prevalence changes were particularly apparent in obesity in men in their 30s (by 10.1%p; 95% CI, 3.6 to 16.5), in hypertension in men in their 50s (by 7.0%p; 95% CI, 1.9 to 12.1), and in hypercholesterolemia in men in their 40s (by 7.7%p; 95% CI, 1.9 to 13.5). In fact, in men, the observed prevalence of the major four chronic diseases in 2020 were at least 2 percentage points higher than the expected prevalence which was calculated using the 2011-2019 data (data not shown). In women, the prevalence of obesity, high blood pressure, diabetes, and hypercholesterolemia in 2020 was not significantly different from the 2017-2019 average. However, among women in their 20s, the prevalence of obesity significantly increased in 2020 compared to the last 3 years (by 5.8%p; 95% CI, 0.4 to 11.2). Additionally, in 2020, women had constant or decreased prevalence in most chronic diseases, except for obesity, compared to the expected prevalence (data not shown).

Overall, people with lower income levels tended to have a higher prevalence of obesity, hypertension, and diabetes than those with higher income levels, and the discrepancies have been widening over time (Supplementary Material 6). The prevalence of hypercholesterolemia rapidly increased in all income groups, but income-related disparities have not yet been observed (Supplementary Materials 6). Notably, in 2020 compared to the last 3 years, significant increases in obesity (by 4.8%p; 95% CI, 0.7 to 8.9) and diabetes (by 3.4%p; 95% CI, 0.6 to 6.1) were found in the low-income group (Table 2).

## DISCUSSION

The current study observed secular trends in the prevalence of obesity, hypertension, diabetes, and hypercholesterolemia using Korean national representative data over 10 years. In particular, we compared the 2020 data with the previous three-year average data to confirm the change in the prevalence of chronic diseases before and during the outbreak of COVID-19. Overall, the prevalence of obesity and hypercholesterolemia significantly increased over the last 10 years, and this trend was more prominent in men. Notably, in men, the prevalence of the 3 major chronic diseases (obesity, diabetes, and hypercholesterolemia) increased significantly in 2020 compared to the last 3 years before the COVID-19 pandemic. The prevalence of obesity and diabetes also increased sharply in low-income groups in 2020.

Monitoring the changes in the prevalence of chronic diseases in 2020 is crucial since the sudden outbreak of COVID-19 has led to drastic changes in overall lifestyles. Governments have imposed interventions including isolation to contain viral transmission [18]. However, unintended consequences also have been observed, such as economic crises and deterioration of health behaviors [19,20]. These consequences may result in long-lasting effects on cardio-metabolic health [21]. By updating the 2020 results using national health examination data, as expected, we observed overall increases in the prevalence of obesity, hypertension, diabetes, and hypercholesterolemia. The strict implementation of physical distancing in the private and public sectors led to increases in involuntary physical inactivity and sedentary behaviors, which have been well described as CVD risk factors [22-24]. Many companies have encouraged telecommuting, and people have spent more time at home. Decreased frequencies of meaningful social interactions [25] or experiences of disease outbreaks [26] may also have led to a deterioration of psychological health in many individuals. Psychological stress is related to an increased risk of developing obesity through altered food-related behaviors and hormone secretions [27]. Interestingly, in men, the prevalence of chronic diseases tended to rise. Since a higher proportion of men than women engage in economic activity [28], there may have been more psychological stress or changes in the working environment during the COVID-19 pandemic.

We observed greater increases in the prevalence of obesity and diabetes in 2020 than in the previous 3 years among the lowest-income group than among the higher-income groups. Economic hardships often worsen the circumstances of the poorest people in society and thereby exacerbate socioeconomic inequality. People with lower socioeconomic status are more likely to preferentially select cheap and highly processed food due to their limited income and resources [15]. Moreover, CVD risk-related problems in health behaviors, psychological health, and access to primary care among deprived people have been well-described in previous studies [29-31].

Inconsistent results have been reported regarding health metrics during the COVID-19 outbreak. Several studies reported improvements in blood pressure or glucose levels among patients with hypertension or diabetes after the COVID-19 lockdown [19,32]. In contrast, some studies reported an increased obesity prevalence [8,33,34]. However, these previous studies cannot be generalized due to their small sample sizes, as well as the use of samples consisting of medical patients [19,32,35] or minority communities [34]. Additionally, most studies reporting increased obesity during the pandemic utilized self-reported BMI [8,33]. Instead, a strength of our study is that it utilized nationally representative Korean data with objectively measured outcomes obtained using standardized protocols. However, several limitations need to be considered. First, although the KNHANES was designed to represent the Korean population, the sample was limited to the non-institutionalized civilian population. Therefore, people who were admitted to hospitals or nursing homes were not included in the current study. Second, since the information on disease diagnosis and medication usage was obtained through self-reporting, there remains a possibility of measurement error. Lastly, the sample size may not have been sufficient to determine the management status of chronic diseases. One round of the KNHANES is designed to be completed by combining 3 years of data. We calculated the prevalence of chronic diseases by year, but could not calculate the management indicators by year because of an insufficient sample size. In addition, although there was an increase in chronic diseases in 2020, the possibility cannot be ruled out that the increase was observed by chance due to random error or variation.

## CONCLUSION

Over the last 10 years, significant increases in obesity and hypercholesterolemia have been prominent among the Korean population. Notably, during the COVID-19 pandemic, increases in the prevalence of obesity, diabetes, and hypercholesterolemia were observed in the Korean men adult population. Moreover, people with lower income tended to show larger increases in the prevalence of obesity and diabetes in 2020 than higher-income groups. Our findings may have important public health implications: the COVID-19 pandemic may have long-term adverse impacts on the cardiovascular and metabolic health of the entire population, regardless of COVID-19 infection, but the impact may not be the same for everyone. It is also necessary to study which chronic diseases are more serious and which prevention strategies are more effective for each target group.

## Figures and Tables

**Figure f2-epih-44-e2022041:**
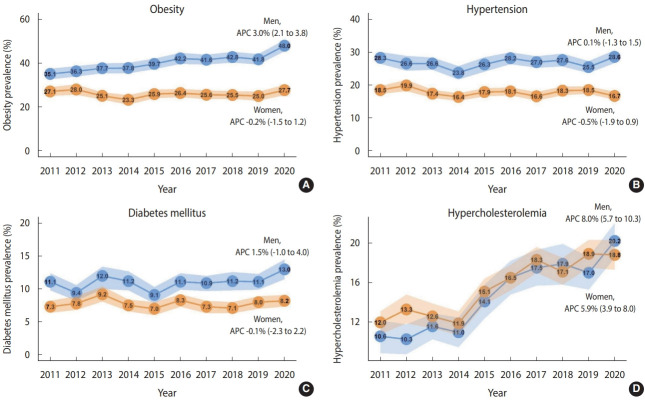


**Table 1.** General characteristics of the participants in the KNHANES

Characteristics		2011	2012	2013	2014	2015	2016	2017	2018	2019	2020
Men		2,651 (49.4)	2,506 (49.3)	2,485 (49.6)	2,385 (49.4)	2,457 (49.4)	2,647 (49.7)	2,749 (49.8)	2,736 (49.9)	2,795 (49.8)	2,651 (49.7)
Mean age (yr)		45.5 (44.7, 46.3)	45.7 (45.0, 46.4)	46.0 (44.7, 46.3)	45.5 (44.7, 46.3)	45.5 (44.7, 46.3)	45.5 (44.7, 46.3)	45.5 (44.7-46.3)	45.5 (44.7-46.3)	45.5 (44.7-46.3)	45.5 (44.7-46.3)
	19-29	699 (19.0)	664 (18.8)	728 (18.5)	638 (18.6)	693 (18.4)	701 (17.7)	728 (17.5)	764 (17.8)	753 (17.5)	801 (17.3)
	30-39	1,125 (20.7)	1,017 (20.2)	1,024 (19.7)	965 (19.1)	769 (18.6)	1,089 (18.3)	915 (17.9)	916 (17.4)	929 (17.1)	769 (16.5)
	40-49	1,089 (21.9)	1,022 (21.5)	1,137 (21.2)	981 (20.9)	987 (20.6)	1,143 (20.8)	1,138 (20.4)	1,134 (19.9)	1,125 (19.3)	973 (19.0)
	50-59	1,212 (18.1)	1,137 (18.7)	1,096 (19.2)	1,082 (19.5)	1,164 (19.7)	1,110 (19.9)	1,215 (19.9)	1,198 (19.8)	1,186 (19.9)	1,065 (19.7)
	60-69	1,048 (10.6)	1,054 (10.7)	907 (10.9)	974 (11.2)	1,026 (11.8)	1,019 (12.4)	1,101 (12.9)	1,109 (13.4)	1,135 (14.1)	1,124 (14.9)
	≥70	1,051 (9.8)	1,101 (10.1)	900 (10.5)	1,037 (10.7)	993 (10.9)	1,067 (10.9)	1,096 (11.3)	1,117 (11.7)	1,168 (12.1)	1,190 (12.5)
Household income											
	Low	1,246 (23.1)	1,175 (22.4)	1,147 (20.0)	1,142 (20.9)	1,105 (20.2)	1,222 (20.8)	1,230 (19.6)	1,245 (20.9)	1,260 (19.6)	1,170 (19.5)
	Low-middle	1,215 (20.5)	1,193 (20.7)	1,140 (20.0)	1,122 (20.1)	1,122 (20.1)	1,228 (19.4)	1,234 (20.3)	1,245 (20.8)	1,248 (20.0)	1,176 (19.3)
	Middle	1,220 (19.8)	1,151 (20.0)	1,164 (20.4)	1,128 (19.5)	1,128 (20.4)	1,222 (20.0)	1,231 (19.3)	1,255 (20.5)	1,246 (19.9)	1,182 (20.1)
	Middle-high	1,226 (18.8)	1,181 (18.4)	1,153 (20.2)	1,138 (19.7)	1,118 (19.8)	1,220 (19.7)	1,236 (20.7)	1,232 (19.3)	1,255 (20.3)	1,184 (20.6)
	High	1,246 (17.8)	1,196 (18.4)	1,142 (19.5)	1,116 (19.8)	1,118 (19.6)	1,213 (20.0)	1,237 (20.0)	1,240 (18.5)	1,253 (20.2)	1,183 (20.5)
Body mass index (kg/m^2^)		23.7 (23.5, 23.8)	23.8 (23.6, 23.9)	23.7 (23.6, 23.8)	23.6 (23.5, 23.8)	23.9 (23.8, 24.0)	24.0 (23.9, 24.1)	23.9 (23.8, 24.0)	24.0 (23.9, 24.1)	23.9 (23.8, 24.1)	24.3 (24.2, 24.4)
Systolic blood pressure (mmHg)		117.6 (116.9, 118.2)	117.8 (117.1, 118.6)	116.6 (116.0, 117.3)	116.3 (115.6, 117.0)	117.4 (116.8, 118.1)	118.1 (117.4, 118.7)	117.4 (116.7, 118.1)	117.7 (117.0, -118.4)	118.4 (117.8, 119.1)	118.3 (117.5, 119)
Diastolic blood pressure (mmHg)		76.0 (75.7, 76.4)	75.9 (75.4, 76.4)	75.0 (74.6, 75.5)	74.8 (74.3, 75.3)	75.1 (74.7, 75.5)	75.9 (75.5, 76.3)	75.7 (75.3, 76.1)	75.9 (75.5, 76.3)	76.0 (75.6, 76.3)	76.1 (75.7, 76.5)
Hemoglobin A1c (%)		5.7 (5.7, 5.7)	5.7 (5.7, 5.7)	5.8 (5.8, 5.9)	5.7 (5.7, 5.7)	5.6 (5.6, 5.7)	5.6 (5.6, 5.7)	5.6 (5.6, 5.7)	5.7 (5.6, 5.7)	5.7 (5.7, 5.8)	5.8 (5.7, 5.8)
Fasting blood glucose (mg/dL)		96.6 (95.9, 97.3)	97.3 (96.5, 98.1)	98.5 (97.8, 99.3)	99.0 (98.2, 99.7)	100.0 (99.1, 100.8)	100.4 (99.5, 101.3)	99.7 (98.9, 100.5)	100.2 (99.5, 100.9)	100.4 (99.6, 101.2)	101.0 (100.2, 101.8)
Total cholesterol (mg/dL)		188.6 (187.2, 190.1)	188.7 (187.3, 190.1)	187.3 (185.9, 188.6)	186.6 (185.3, 188.0)	190.0 (188.9, 191.1)	193.1 (191.9, 194.4)	193.3 (192.0, 194.6)	191.8 (190.5, 193.1)	193.0 (191.9, 194.1)	190.9 (189.7, 192.1)
Triglyceride (mg/dL)		133.3 (129.5, 137.1)	134.6 (130.0, 139.2)	138.1 (134.1, 142.2)	138.1 (134.1, 142.1)	139.0 (134.7, 143.4)	144.8 (139.3, 150.4)	135.9 (132.4, 139.5)	136.3 (132.7, 139.8)	132.1 (128.8, 135.3)	136.8 (132.4, 141.1)
LDL-cholesterol (mg/dL)		112.7 (110.8, 114.5)	121.0 (118.0, 123.9)	113.7 (111.0, 116.5)	117.4 (114.2, 120.7)	113.7 (112.6, 114.8)	117.4 (114.9, 119.8)	121.4 (118.4, 124.5)	118.0 (115.3, 120.8)	118.6 (115.8, 121.5)	113.9 (110.9, 117.0)
HDL-cholesterol (mg/dL)		53.1 (52.6, 53.6)	51.7 (51.1, 52.2)	52.3 (51.8, 52.7)	52.6 (52.2, 53.0)	53.8 (53.4, 54.2)	53.7 (53.3, 54.2)	51.9 (51.4, 52.3)	52.7 (52.2, 53.2)	52.8 (52.4, 53.3)	51.4 (51.0, 51.9)

Values are presented as number or mean (weighted %, 95% confidence interval). KNHANES, Korea National Health and Nutritional Examination Survey; HDL, high-density lipoprotein; LDL, low-density lipoprotein.

**Table 2.** Changes in the prevalence of obesity, hypertension, diabetes mellitus, and hypercholesterolemia in 2020

Category		Obesity prevalence			Hypertension prevalence			Diabetes mellitus prevalence			Hypercholesterolemia prevalence		
		2017-2019	2020	Changes	2017-2019	2020	Changes	2017-2019	2020	Changes	2017-2019	2020	Changes
				%p (95% CI)			%p (95% CI)			%p (95% CI)			%p (95% CI)
Total, age ≥19		34.2	38.4	4.2 (2.2, 6.2)	22.5	22.9	0.4 (-1.1, 1.9)	9.3	10.7	1.4 (0.3, 2.4)	18.0	19.7	1.7 (0.3, 3.1)
Men, age ≥19		42.1	48.0	6.0 (3.2, 8.8)	26.7	28.6	1.8 (-0.5, 4.0)	11.1	13.0	1.9 (0.3, 3.5)	17.4	20.2	2.8 (0.8, 4.9)
	19-29	37.5	41.5	4.0 (-1.8, 9.8)	8.0	7.4	-0.6 (-4.3, 3.0)	1.5	0.6	-0.9 (-2.1, 0.3)	6.4	5.3	-1.1 (-4.2, 1.9)
	30-39	48.2	58.2	10.1 (3.6, 16.5)	16.7	17.7	1.0 (-4.0, 5.9)	3.4	5.9	2.5 (-0.4, 5.4)	14.2	12.7	-1.4 (-5.9, 3.1)
	40-49	45.7	50.6	4.9 (-1.4, 11.1)	27.5	31.5	4.0 (-1.5, 9.5)	11.3	14.1	2.8 (-1.6, 7.2)	20.5	28.2	7.7 (1.9, 13.5)
	50-59	42.9	48.3	5.4 (-0.1, 10.8)	38.4	45.4	7.0 (1.9, 12.1)	19.1	23.8	4.7 (-0.2, 9.6)	23.9	27.2	3.3 (-1.6, 8.2)
	60-69	38.3	44.1	5.8 (0.1, 11.5)	49.2	50.5	1.2 (-4.7, 7.2)	25.6	28.8	3.2 (-2.1, 8.6)	28.4	35.4	6.9 (1.2, 12.6)
	≥70	28.8	32.4	3.6 (-2.3, 9.4)	62.0	59.3	-2.8 (-8.1, 2.6)	29.7	28.7	-0.9 (-6.5, 4.7)	25.3	32.4	7.1 (0.6, 13.6)
Women, age ≥19		25.3	27.8	2.5 (-0.1, 5.0)	17.8	16.8	-1.1 (-2.5, 0.4)	7.5	8.2	0.8 (-0.3, 1.9)	18.2	18.8	0.7 (-1.0, 2.4)
	19-29	17.0	22.8	5.8 (0.4, 11.2)	2.1	1.1	-1.0 (-2.5, 0.4)	1.2	0.8	-0.4 (-1.6, 0.8)	3.6	5.0	1.4 (-1.2, 4.1)
	30-39	20.8	22.7	1.9 (-3.5, 7.2)	4.5	4.4	-0.1 (-2.4, 2.2)	2.0	2.7	0.7 (-1.1, 2.6)	8.1	8.2	0.2 (-3.0, 3.4)
	40-49	25.7	27.1	1.4 (-3.9, 6.7)	11.4	9.6	-1.8 (-4.9, 1.3)	4.5	3.7	-0.8 (-3.0, 1.3)	13.6	13.8	0.2 (-3.9, 4.4)
	50-59	30.2	32.4	2.2 (-2.8, 7.1)	27.6	26.4	-1.2 (-5.6, 3.2)	11.0	14.3	3.3 (-0.8, 7.4)	33.4	31.7	-1.7 (-6.7, 3.4)
	60-69	36.5	38.7	2.2 (-3.0, 7.4)	47.3	45.9	-1.4 (-6.8, 4.0)	19.7	21.6	1.9 (-2.5, 6.3)	46.3	48.0	1.6 (-4.2, 7.5)
	≥70	40.3	38.1	-2.2 (-7.1, 2.6)	71.0	69.6	-1.5 (-6.2, 3.3)	30.6	33.5	2.9 (-1.7, 7.5)	42.6	46.0	3.4 (-2.6, 9.5)
Household income													
	Low	36.3	41.2	4.8 (0.7, 8.9)	24.9	24.9	-0.4 (-3.6, 2.9)	11.4	14.8	3.4 (0.6, 6.1)	17.8	19.9	2.1 (-1.0, 5.2)
	Low-middle	35.9	40.0	4.1 (0.0, 8.2)	24.0	24.0	1.0 (-2.3, 4.4)	10.3	11.4	1.1 (-0.9, 3.2)	18.5	19.5	1.0 (-2.0, 4.0)
	Middle	34.0	39.6	5.6 (1.6, 9.7)	21.5	21.5	-0.3 (-3.2, 2.6)	8.8	10.6	1.8 (-0.4, 4.0)	18.3	18.9	0.6 (-2.3, 3.5)
	Middle-high	34.1	38.1	4.0 (0.1, 8.0)	20.9	20.9	2.0 (-1.0, 5.0)	8.3	8.9	0.5 (-1.2, 2.3)	17.2	18.8	1.6 (-1.2, 4.4)
	High	30.2	33.4	3.2 (-0.9, 7.4)	21.1	21.1	-0.3 (-3.3, 2.8)	7.7	8.1	0.4 (-1.2, 2.1)	18.0	21.5	3.4 (0.3, 6.6)

Values are presented as weighted %. Age-standardized prevalence was calculated using the 2005 population projections for Korea.

## SUPPLEMENTARY MATERIALS

Supplementary Material 1. Prevalence of obesity (body mass index≥25kg/m^2^) by sex and age using the Korea National Health and Nutrition Examination Survey (KNHANES) from 2011 to 2020^1^

Supplementary Material 2 Prevalence of hypertension by sex and age using the Korea National Health and Nutrition Examination Survey (KNHANES) from 2011 to 2020^1^

Supplementary Material 3 Prevalence of diabetes mellitus by sex and age using the Korea National Health and Nutrition Examination Survey (KNHANES) from 2011 to 2020^1^

Supplementary Material 4 Prevalence of hypercholesterolemia by sex and age using the Korea National Health and Nutrition Examination Survey (KNHANES) from 2011 to 2020^1^

Supplementary Material 5 Prevalence of severe obesity (body mass index≥30kg/m2) by sex and age using the Korea National Health and Nutrition Examination Survey (KNHANES) from 2011 to 2020^1^

Supplementary Material 6 Prevalence by income level in obesity, hypertension, diabetes mellitus, and hypercholesterolemia.
