# Supplementary material for: Obesity, hypertension, diabetes mellitus, and hypercholesterolemia in Korean adults before and during the COVID-19 pandemic: a special report of the 2020 Korea National Health and Nutrition Examination Survey
Source: Epidemiol Health. 2022 Apr 25;44:e2022041. doi: 10.4178/epih.e2022041 (PMC9133598; doi:10.4178/epih.e2022041)
Supplement: Supplementary Material 1. — Prevalence of obesity (body mass index≥25kg/m2) by sex and age using the Korea National Health and Nutrition Examination Survey (KNHANES) from 2011 to 20201 [file epih-44-e2022041-suppl1.docx]

| Supplementary Material 1. Prevalence of obesity (body mass index≥25kg/m^2^) by sex and age using the Korea National Health and Nutrition Examination Survey (KNHANES) from 2011 to 2020^1^ | | | | | | | | | | | | | | | | | | | | | | | | | |
| --- | --- | --- | --- | --- | --- | --- | --- | --- | --- | --- | --- | --- | --- | --- | --- | --- | --- | --- | --- | --- | --- | --- | --- | --- | --- |
| Characteristics | 2011 | | 2012 | | 2013 | | 2014 | | 2015 | | 2016 | | 2017 | | 2018 | | 2019 | | 2020 | | Annual Percent Change | | | |  |
| Total, age≥19 | 31.4 | (29.7;33.1) | 32.4 | (30.7;34.2) | 31.8 | (30.4;33.2) | 30.9 | (29.2;32.6) | 33.2 | (31.5;34.8) | 34.8 | (33.1;36.5) | 34.1 | (32.4;35.8) | 34.6 | (33.0;36.3) | 33.8 | (32.3;35.4) | 38.4 | (36.7;40.1) | 1.8* | (0.8 | ; | 2.8) |  |
| 19-29 | 21.7 | (17.8;25.7) | 22.4 | (18.6;26.2) | 22.4 | (18.9;25.9) | 23.9 | (20.0;27.9) | 23.5 | (19.8;27.1) | 27.2 | (22.9;31.6) | 29.4 | (25.6;33.2) | 26.9 | (23.0;30.8) | 27.6 | (23.9;31.4) | 32.6 | (29.2;35.9) | 4.5* | (3.0 | ; | 6.0) |  |
| 30-39 | 31.5 | (28.0;35.0) | 32.5 | (28.6;36.4) | 33.2 | (29.7;36.7) | 31.8 | (28.6;35.1) | 32.9 | (29.0;36.8) | 34.2 | (31.0;37.3) | 33.4 | (29.7;37.1) | 37.8 | (33.8;41.8) | 34.9 | (31.3;38.5) | 41.6 | (37.3;46.0) | 2.4* | (1.0 | ; | 3.8) |  |
| 40-49 | 35.4 | (31.9;38.8) | 39.2 | (36.2;42.3) | 33.7 | (30.7;36.8) | 31.1 | (28.0;34.2) | 35.6 | (32.1;39.2) | 39.0 | (35.8;42.3) | 35.3 | (31.5;39.1) | 36.8 | (33.4;40.2) | 35.6 | (32.6;38.5) | 39.1 | (35.3;42.9) | 0.4 | (-1.5 | ; | 2.3) |  |
| 50-59 | 35.7 | (32.4;39.0) | 34.0 | (30.4;37.6) | 37.3 | (34.0;40.6) | 35.4 | (32.5;38.4) | 38.3 | (35.1;41.4) | 36.1 | (32.5;39.7) | 38.0 | (34.9;41.1) | 35.2 | (32.0;38.3) | 36.5 | (33.5;39.5) | 40.3 | (36.9;43.7) | 0.9 | (-0.3 | ; | 2.0) |  |
| 60-69 | 38.8 | (34.9;42.6) | 38.5 | (34.6;42.4) | 36.3 | (32.5;40.1) | 36.8 | (33.5;40.0) | 40.1 | (36.3;43.8) | 40.2 | (36.4;44.0) | 38.0 | (34.9;41.1) | 36.8 | (33.5;40.1) | 37.3 | (34.0;40.6) | 41.3 | (37.7;45.0) | 0.3 | (-0.8 | ; | 1.5) |  |
| 70+ | 29.7 | (26.3;33.1) | 31.1 | (27.1;35.0) | 33.8 | (29.8;37.8) | 32.1 | (28.8;35.5) | 37.4 | (33.7;41.1) | 37.5 | (34.1;41.0) | 34.7 | (31.6;37.8) | 38.0 | (34.3;41.6) | 34.3 | (30.8;37.8) | 35.7 | (32.6;38.9) | 1.8* | (0.1 | ; | 3.5) |  |
|  |  |  |  |  |  |  |  |  |  |  |  |  |  |  |  |  |  |  |  |  |  |  |  |  |  |
| Men, age≥19 | 35.1 | (32.7;37.5) | 36.3 | (33.9;38.7) | 37.7 | (35.4;39.9) | 37.8 | (35.4;40.2) | 39.7 | (37.3;42.1) | 42.2 | (39.7;44.8) | 41.6 | (39.4;43.9) | 42.8 | (40.3;45.2) | 41.8 | (39.4;44.2) | 48.0 | (45.5;50.6) | 3.0* | (2.1 | ; | 3.8) |  |
| 19-29 | 26.2 | (20.5;32.0) | 30.5 | (24.8;36.2) | 29.3 | (24.0;34.7) | 32.0 | (25.4;38.6) | 32.4 | (26.7;38.2) | 38.8 | (32.1;45.6) | 39.0 | (33.3;44.7) | 36.1 | (30.2;42.1) | 37.3 | (32.0;42.7) | 41.5 | (36.5;46.4) | 4.4* | (2.8 | ; | 6.0) |  |
| 30-39 | 40.7 | (35.2;46.2) | 40.6 | (34.7;46.5) | 47.1 | (41.6;52.6) | 43.9 | (39.0;48.8) | 43.6 | (37.7;49.5) | 45.3 | (40.2;50.4) | 46.7 | (41.5;52.0) | 51.3 | (45.7;57.0) | 46.4 | (41.3;51.6) | 58.2 | (52.3;64.1) | 3.0* | (1.2 | ; | 4.9) |  |
| 40-49 | 42.6 | (37.6;47.6) | 45.0 | (39.9;50.1) | 41.5 | (36.7;46.3) | 39.6 | (34.7;44.5) | 45.6 | (40.1;51.2) | 49.0 | (44.5;53.5) | 44.7 | (39.5;49.9) | 47.5 | (42.6;52.4) | 45.0 | (40.4;49.6) | 50.6 | (44.9;56.3) | 1.6* | (0.0 | ; | 3.1) |  |
| 50-59 | 34.7 | (29.5;39.9) | 33.2 | (27.7;38.7) | 40.8 | (36.1;45.5) | 41.5 | (36.8;46.3) | 40.3 | (35.5;45.1) | 39.7 | (34.3;45.1) | 44.3 | (39.8;48.9) | 40.9 | (35.9;46.0) | 43.4 | (38.7;48.1) | 48.3 | (43.6;52.9) | 2.9* | (1.3 | ; | 4.5) |  |
| 60-69 | 34.1 | (28.4;39.8) | 33.5 | (27.9;39.1) | 29.3 | (24.1;34.5) | 36.9 | (32.1;41.8) | 38.3 | (33.0;43.6) | 39.7 | (34.0;45.4) | 36.7 | (31.4;41.9) | 38.1 | (33.3;43.0) | 39.9 | (34.7;45.2) | 44.1 | (39.2;49.0) | 3.0* | (1.3 | ; | 4.7) |  |
| 70+ | 23.7 | (18.4;28.9) | 23.0 | (18.0;28.0) | 26.2 | (21.0;31.5) | 24.0 | (19.3;28.7) | 32.1 | (26.2;37.9) | 30.3 | (25.9;34.8) | 25.3 | (21.1;29.5) | 30.6 | (25.1;36.1) | 30.4 | (25.1;35.6) | 32.4 | (27.1;37.6) | 3.3* | (0.8 | ; | 6.0) |  |
|  |  |  |  |  |  |  |  |  |  |  |  |  |  |  |  |  |  |  |  |  |  |  |  |  |  |
| Women, age≥19 | 27.1 | (25.1;29.1) | 28.0 | (25.6;30.3) | 25.1 | (23.4;26.7) | 23.3 | (21.1;25.4) | 25.9 | (24.0;27.9) | 26.4 | (24.5;28.3) | 25.6 | (23.6;27.6) | 25.5 | (23.7;27.3) | 25.0 | (22.9;27.0) | 27.8 | (25.4;30.1) | -0.2 | (-1.5 | ; | 1.2) |  |
| 19-29 | 16.9 | (12.2;21.5) | 13.6 | (9.5;17.7) | 14.4 | (10.7;18.1) | 15.0 | (10.8;19.1) | 13.4 | (9.4;17.4) | 13.8 | (10.1;17.6) | 18.3 | (13.7;23.0) | 16.2 | (11.8;20.6) | 16.5 | (11.9;21.1) | 22.8 | (18.0;27.6) | 3.9* | (0.2 | ; | 7.7) |  |
| 30-39 | 21.7 | (17.5;25.9) | 23.7 | (18.8;28.7) | 17.9 | (14.8;21.0) | 18.6 | (14.6;22.5) | 21.1 | (16.9;25.3) | 21.7 | (18.0;25.3) | 18.3 | (14.3;22.4) | 22.6 | (18.2;26.9) | 21.6 | (16.9;26.4) | 22.7 | (18.0;27.4) | 0.7 | (-1.9 | ; | 3.4) |  |
| 40-49 | 27.9 | (24.0;31.8) | 33.2 | (28.7;37.7) | 25.7 | (21.6;29.8) | 22.3 | (18.0;26.5) | 25.4 | (21.1;29.7) | 28.7 | (24.4;33.0) | 25.6 | (21.2;30.1) | 25.7 | (21.9;29.6) | 25.8 | (21.9;29.7) | 27.1 | (22.2;31.9) | -1.3 | (-3.8 | ; | 1.3) |  |
| 50-59 | 36.7 | (32.6;40.9) | 34.9 | (30.3;39.5) | 33.7 | (29.5;37.9) | 29.3 | (25.5;33.1) | 36.2 | (31.9;40.5) | 32.5 | (28.0;37.0) | 31.7 | (27.5;35.8) | 29.3 | (25.3;33.4) | 29.6 | (25.8;33.5) | 32.4 | (28.0;36.7) | -1.8 | (-3.5 | ; | 0.0) |  |
| 60-69 | 43.0 | (37.9;48.1) | 43.1 | (37.8;48.4) | 42.7 | (37.4;48.0) | 36.6 | (32.3;41.0) | 41.7 | (36.8;46.6) | 40.7 | (36.0;45.4) | 39.3 | (35.0;43.5) | 35.5 | (31.1;39.8) | 34.9 | (30.6;39.1) | 38.7 | (34.1;43.2) | -1.9 | (-3.3 | ; | -0.4) |  |
| 70+ | 33.5 | (29.3;37.7) | 36.1 | (31.1;41.1) | 38.6 | (33.5;43.7) | 37.3 | (33.0;41.6) | 40.8 | (36.2;45.4) | 42.2 | (37.4;47.0) | 41.0 | (36.7;45.3) | 43.0 | (38.7;47.2) | 37.0 | (32.8;41.2) | 38.1 | (33.9;42.2) | 1.3 | (-0.6 | ; | 3.2) |  |
| Household income | |  |  |  |  |  |  |  |  |  |  |  |  |  |  |  |  |  |  |  |  |  |  |  |  |
| Low | 32.2 | (28.5;35.9) | 33.7 | (29.7;37.6) | 32.1 | (29.3;35.0) | 33.2 | (29.6;36.8) | 38.4 | (34.7;42.2) | 38.4 | (34.3;42.4) | 38.5 | (34.9;42.0) | 35.1 | (31.6;38.6) | 35.6 | (32.3;38.8) | 41.2 | (37.6;44.7) | 2.2* | (0.6 | ; | 3.9) |  |
| Low-middle | 31.9 | (27.9;35.9) | 37.2 | (33.2;41.1) | 34.2 | (30.6;37.7) | 33.0 | (29.3;36.7) | 34.1 | (30.8;37.3) | 33.4 | (29.9;36.9) | 35.8 | (32.7;38.9) | 38.4 | (34.9;41.9) | 33.6 | (30.3;37.0) | 40.0 | (36.5;43.6) | 1.4 | (-0.3 | ; | 3.1) |  |
| Middle | 33.8 | (30.0;37.6) | 31.3 | (27.1;35.5) | 30.4 | (27.4;33.4) | 33.1 | (29.6;36.6) | 32.2 | (28.4;35.9) | 35.4 | (32.0;38.7) | 32.3 | (28.5;36.0) | 35.4 | (31.9;38.9) | 34.1 | (30.8;37.5) | 39.6 | (36.1;43.1) | 1.9* | (0.4 | ; | 3.5) |  |
| Middle-high | 31.0 | (27.2;34.8) | 29.9 | (25.9;33.9) | 30.7 | (27.4;34.0) | 30.4 | (26.8;34.0) | 32.0 | (28.0;35.9) | 33.6 | (30.1;37.1) | 33.8 | (30.1;37.4) | 35.5 | (32.0;38.9) | 33.1 | (30.0;36.2) | 38.1 | (34.7;41.6) | 2.3* | (1.3 | ; | 3.4) |  |
| High | 27.4 | (23.7;31.2) | 30.3 | (25.9;34.7) | 31.3 | (28.0;34.6) | 24.8 | (21.8;27.8) | 30.1 | (26.5;33.6) | 33.3 | (29.9;36.7) | 30.6 | (27.3;33.9) | 28.1 | (24.6;31.5) | 31.8 | (27.8;35.7) | 33.4 | (30.0;36.9) | 1.5 | (-0.8 | ; | 3.8) |  |
| Values are presented as weighted % (95% confidence interval). Age-standardized prevalence was calculated using the 2005 Population Projections for Korea.  *The annual percent change (APC) is significantly different from 0. | | | | | | | | | | | | | | | | | | | | | | | | | |
