# Supplementary material for: Obesity, hypertension, diabetes mellitus, and hypercholesterolemia in Korean adults before and during the COVID-19 pandemic: a special report of the 2020 Korea National Health and Nutrition Examination Survey
Source: Epidemiol Health. 2022 Apr 25;44:e2022041. doi: 10.4178/epih.e2022041 (PMC9133598; doi:10.4178/epih.e2022041)
Supplement: Supplementary Material 2 — Prevalence of hypertension by sex and age using the Korea National Health and Nutrition Examination Survey (KNHANES) from 2011 to 20201 [file epih-44-e2022041-suppl2.docx]

| Supplementary Material 2. Prevalence of hypertension by sex and age using the Korea National Health and Nutrition Examination Survey (KNHANES) from 2011 to 2020^1^ | | | | | | | | | | | | | | | | | | | | | | | | |
| --- | --- | --- | --- | --- | --- | --- | --- | --- | --- | --- | --- | --- | --- | --- | --- | --- | --- | --- | --- | --- | --- | --- | --- | --- |
| Characteristics | 2011 | | 2012 | | 2013 | | 2014 | | 2015 | | 2016 | | 2017 | | 2018 | | 2019 | | 2020 | | Annual Percent Change | | | |
| Total, age≥19 | 23.6 | (22.4;24.7) | 23.4 | (21.9;24.9) | 22.0 | (20.8;23.3) | 20.1 | (18.9;21.4) | 22.1 | (20.9;23.4) | 23.3 | (22.2;24.4) | 22.0 | (20.8;23.3) | 23.1 | (21.8;24.5) | 22.2 | (21.1;23.3) | 22.9 | (21.6;24.2) | -0.1 | (-1.3 | ; | 1.2) |
| 19-29 | 7.0 | (4.7;9.3) | 4.8 | (2.3;7.2) | 4.2 | (2.6;5.9) | 2.2 | (0.7;3.7) | 2.7 | (1.6;3.9) | 3.5 | (1.9;5.1) | 5.1 | (2.7;7.5) | 5.4 | (3.6;7.3) | 5.2 | (3.4;7.1) | 4.4 | (2.7;6.1) | -1.1 | (-8.3 | ; | 6.6) |
| 30-39 | 9.1 | (7.2;11.0) | 9.5 | (6.9;12.1) | 9.7 | (7.5;12.0) | 7.3 | (5.6;9.0) | 8.7 | (6.2;11.3) | 10.3 | (8.2;12.4) | 11.3 | (9.0;13.5) | 11.7 | (9.1;14.2) | 9.5 | (7.3;11.8) | 11.4 | (8.9;13.9) | 2.8 | (-0.3 | ; | 5.9) |
| 40-49 | 21.1 | (18.0;24.1) | 22.6 | (19.2;26.1) | 19.5 | (16.9;22.2) | 17.7 | (14.8;20.6) | 20.7 | (17.5;24.0) | 21.7 | (19.0;24.4) | 19.2 | (16.4;22.0) | 20.6 | (17.8;23.4) | 19.0 | (16.4;21.5) | 20.8 | (17.9;23.7) | -0.5 | (-2.2 | ; | 1.3) |
| 50-59 | 33.8 | (30.6;37.0) | 34.5 | (30.8;38.1) | 35.9 | (32.2;39.5) | 32.0 | (28.4;35.7) | 33.9 | (30.6;37.1) | 36.5 | (33.4;39.7) | 32.1 | (29.1;35.2) | 34.7 | (31.5;37.8) | 32.3 | (28.9;35.6) | 35.9 | (32.7;39.0) | 0.1 | (-1.2 | ; | 1.4) |
| 60-69 | 55.4 | (51.3;59.4) | 54.0 | (50.0;57.9) | 48.7 | (44.3;53.0) | 48.5 | (44.6;52.4) | 51.8 | (47.8;55.8) | 50.9 | (47.3;54.5) | 46.9 | (43.2;50.6) | 46.0 | (42.7;49.4) | 51.5 | (47.9;55.1) | 48.1 | (44.2;52.0) | -1.2 | (-2.5 | ; | 0.0) |
| 70+ | 66.6 | (62.6;70.6) | 66.4 | (63.2;69.5) | 62.3 | (57.5;67.0) | 63.5 | (59.4;67.5) | 67.5 | (63.9;71.2) | 69.2 | (66.2;72.2) | 64.7 | (61.1;68.4) | 70.2 | (67.2;73.3) | 67.2 | (64.0;70.4) | 65.3 | (62.2;68.5) | 0.3 | (-0.6 | ; | 1.2) |
|  |  |  |  |  |  |  |  |  |  |  |  |  |  |  |  |  |  |  |  |  |  |  |  |  |
| Men, age≥19 | 28.3 | (26.5;30.1) | 26.6 | (24.4;28.9) | 26.6 | (24.7;28.4) | 23.8 | (21.7;25.8) | 26.3 | (24.1;28.5) | 28.2 | (26.4;30.0) | 27.0 | (25.3;28.8) | 27.6 | (25.7;29.6) | 25.5 | (23.7;27.3) | 28.6 | (26.7;30.6) | 0.1 | (-1.3 | ; | 1.5) |
| 19-29 | 12.6 | (8.4;16.9) | 7.8 | (3.2;12.3) | 6.7 | (3.9;9.5) | 3.3 | (0.6;6.1) | 4.8 | (2.7;6.9) | 5.1 | (2.4;7.7) | 9.0 | (4.6;13.4) | 8.7 | (5.3;12.1) | 6.4 | (3.7;9.2) | 7.4 | (4.4;10.4) | -3.5 | (-10.9 | ; | 4.6) |
| 30-39 | 14.6 | (11.2;18.1) | 15.5 | (10.9;20.1) | 15.8 | (11.7;19.9) | 13.6 | (10.3;16.9) | 15.9 | (11.2;20.7) | 16.9 | (13.2;20.6) | 17.9 | (14.0;21.8) | 17.1 | (12.8;21.4) | 15.1 | (11.3;18.9) | 17.7 | (13.5;21.9) | 1.9 | (-0.1 | ; | 3.8) |
| 40-49 | 31.2 | (26.1;36.3) | 26.9 | (21.9;31.9) | 28.5 | (24.1;32.8) | 26.9 | (21.7;32.1) | 28.4 | (23.2;33.7) | 30.8 | (26.3;35.4) | 26.9 | (23.0;30.9) | 29.1 | (24.8;33.3) | 26.5 | (22.2;30.8) | 31.5 | (26.5;36.5) | 0.1 | (-1.7 | ; | 1.9) |
| 50-59 | 38.0 | (33.5;42.4) | 38.7 | (33.1;44.3) | 41.3 | (36.1;46.6) | 36.8 | (31.9;41.6) | 38.4 | (33.8;43.1) | 42.3 | (37.2;47.3) | 39.4 | (35.3;43.5) | 40.2 | (35.8;44.5) | 35.7 | (30.3;41.1) | 45.4 | (40.9;49.9) | 1.1 | (-0.5 | ; | 2.8) |
| 60-69 | 53.5 | (47.5;59.5) | 55.3 | (49.5;61.2) | 48.5 | (42.0;55.1) | 45.5 | (39.4;51.6) | 52.1 | (46.7;57.5) | 55.9 | (50.3;61.6) | 47.6 | (42.3;52.8) | 47.5 | (43.0;52.1) | 52.3 | (47.4;57.3) | 50.5 | (45.2;55.7) | -0.6 | (-2.3 | ; | 1.1) |
| 70+ | 58.9 | (53.1;64.7) | 58.1 | (52.3;63.8) | 59.0 | (51.4;66.5) | 55.8 | (49.6;62.0) | 61.7 | (55.0;68.3) | 64.2 | (59.2;69.3) | 61.6 | (56.7;66.4) | 65.1 | (60.4;69.9) | 59.5 | (54.5;64.5) | 59.3 | (54.8;63.8) | 0.5 | (-0.7 | ; | 1.8) |
|  |  |  |  |  |  |  |  |  |  |  |  |  |  |  |  |  |  |  |  |  |  |  |  |  |
| Women, age≥19 | 18.5 | (17.3;19.7) | 19.9 | (18.2;21.6) | 17.4 | (16.1;18.6) | 16.4 | (15.2;17.5) | 17.9 | (16.6;19.1) | 18.1 | (16.9;19.3) | 16.6 | (15.3;17.9) | 18.3 | (16.9;19.6) | 18.5 | (17.4;19.6) | 16.8 | (15.5;18.0) | -0.5 | (-1.9 | ; | 0.9) |
| 19-29 | 0.9 | (;0.1;1.9) | 1.6 | (0.1;3.1) | 1.3 | (0.1;2.6) | 0.9 | (0.0;1.9) | 0.5 | (;0.2;1.2) | 1.7 | (0.4;3.0) | 0.8 | (;0.3;1.9) | 1.7 | (0.5;2.8) | 3.9 | (1.4;6.3) | 1.1 | (0.0;2.2) | 11.2 | (-2.5 | ; | 26.7) |
| 30-39 | 3.4 | (1.9;5.0) | 3.2 | (1.5;4.9) | 3.7 | (1.9;5.5) | 1.1 | (0.2;2.0) | 1.6 | (0.4;2.9) | 3.3 | (1.7;4.8) | 4.2 | (2.3;6.0) | 5.8 | (3.5;8.2) | 3.4 | (1.8;5.0) | 4.4 | (2.4;6.4) | 5.1 | (-4.3 | ; | 15.4) |
| 40-49 | 10.8 | (7.8;13.8) | 18.1 | (13.7;22.6) | 10.7 | (8.2;13.3) | 8.8 | (5.8;11.7) | 13.0 | (9.7;16.3) | 12.4 | (9.3;15.4) | 11.3 | (8.3;14.2) | 11.9 | (8.9;14.9) | 11.1 | (8.6;13.7) | 9.6 | (6.9;12.3) | -2.3 | (-6.9 | ; | 2.5) |
| 50-59 | 29.7 | (25.5;33.9) | 30.4 | (25.9;34.8) | 30.6 | (26.7;34.5) | 27.4 | (23.1;31.7) | 29.5 | (25.4;33.7) | 30.8 | (26.7;34.8) | 24.9 | (21.1;28.7) | 29.1 | (24.9;33.2) | 28.8 | (25.0;32.6) | 26.4 | (22.7;30.1) | -1.1 | (-2.7 | ; | 0.4) |
| 60-69 | 57.1 | (52.3;61.9) | 52.8 | (47.4;58.2) | 48.8 | (43.4;54.2) | 51.1 | (46.4;55.8) | 51.5 | (46.2;56.8) | 46.2 | (41.2;51.2) | 46.3 | (41.3;51.4) | 44.6 | (39.9;49.3) | 50.7 | (45.8;55.6) | 45.9 | (41.2;50.6) | -2.0 | (-3.3 | ; | -0.6) |
| 70+ | 71.5 | (66.2;76.7) | 71.6 | (67.4;75.8) | 64.3 | (58.4;70.3) | 68.7 | (63.9;73.5) | 71.3 | (66.5;76.2) | 72.5 | (68.2;76.7) | 66.8 | (61.5;72.1) | 73.7 | (69.5;77.8) | 72.4 | (68.3;76.6) | 69.6 | (65.6;73.5) | 0.2 | (-0.7 | ; | 1.1) |
| Household income | |  |  |  |  |  |  |  |  |  |  |  |  |  |  |  |  |  |  |  |  |  |  |  |
| Low | 23.4 | (20.9;26.0) | 23.9 | (20.8;27.1) | 21.7 | (19.5;23.9) | 21.3 | (18.8;23.8) | 25.2 | (22.7;27.7) | 26.0 | (23.7;28.3) | 24.6 | (21.7;27.5) | 25.1 | (22.3;27.9) | 25.1 | (22.4;27.8) | 24.6 | (21.8;27.3) | 1.3 | (-0.3 | ; | 2.9) |
| Low-middle | 24.3 | (21.7;26.9) | 24.4 | (21.2;27.7) | 22.8 | (19.9;25.7) | 22.1 | (19.8;24.4) | 24.8 | (22.0;27.6) | 21.1 | (18.6;23.7) | 22.2 | (19.9;24.4) | 26.7 | (24.0;29.4) | 22.9 | (20.7;25.1) | 25.0 | (22.0;28.1) | 0.2 | (-1.7 | ; | 2.2) |
| Middle | 26.5 | (23.6;29.4) | 24.4 | (21.5;27.4) | 24.3 | (21.8;26.8) | 19.3 | (16.9;21.7) | 19.6 | (17.4;21.9) | 22.2 | (20.4;24.1) | 21.3 | (18.5;24.1) | 21.7 | (19.4;24.1) | 21.2 | (18.7;23.8) | 21.2 | (18.8;23.5) | -1.9 | (-4.0 | ; | 0.2) |
| Middle-high | 20.7 | (18.4;22.9) | 22.7 | (19.8;25.6) | 20.1 | (17.7;22.6) | 20.1 | (17.6;22.7) | 20.9 | (18.3;23.5) | 24.6 | (21.9;27.2) | 20.3 | (17.8;22.7) | 21.9 | (19.1;24.8) | 20.4 | (17.9;22.8) | 22.9 | (20.3;25.5) | 0.6 | (-1.2 | ; | 2.4) |
| High | 22.2 | (19.8;24.7) | 21.1 | (18.4;23.8) | 21.1 | (18.2;24.1) | 17.9 | (15.4;20.3) | 20.4 | (17.9;22.8) | 22.6 | (20.2;25.1) | 21.5 | (19.1;23.8) | 20.0 | (17.5;22.4) | 21.7 | (19.2;24.1) | 20.8 | (18.1;23.5) | -0.1 | (-1.8 | ; | 1.6) |
| Values are presented as weighted % (95% confidence interval). Age-standardized prevalence was calculated using the 2005 Population Projections for Korea.  *The annual percent change (APC) is significantly different from 0. | | | | | | | | | | | | | | | | | | | | | | | | |
