# Supplementary material for: Obesity, hypertension, diabetes mellitus, and hypercholesterolemia in Korean adults before and during the COVID-19 pandemic: a special report of the 2020 Korea National Health and Nutrition Examination Survey
Source: Epidemiol Health. 2022 Apr 25;44:e2022041. doi: 10.4178/epih.e2022041 (PMC9133598; doi:10.4178/epih.e2022041)
Supplement: Supplementary Material 3 — Prevalence of diabetes mellitus by sex and age using the Korea National Health and Nutrition Examination Survey (KNHANES) from 2011 to 20201 [file epih-44-e2022041-suppl3.docx]

| Supplementary Material 3. Prevalence of diabetes mellitus by sex and age using the Korea National Health and Nutrition Examination Survey (KNHANES) from 2011 to 2020^1^ | | | | | | | | | | | | | | | | | | | | | | | | | |
| --- | --- | --- | --- | --- | --- | --- | --- | --- | --- | --- | --- | --- | --- | --- | --- | --- | --- | --- | --- | --- | --- | --- | --- | --- | --- |
| Characteristics | 2011 | | 2012 | | 2013 | | 2014 | | 2015 | | 2016 | | 2017 | | 2018 | | 2019 | | 2020 | | Annual Percent Change | | | | |
| Total, age≥19 | 9.3 | (8.4;10.1) | 8.6 | (7.7;9.4) | 10.7 | (9.7;11.6) | 9.3 | (8.4;10.2) | 8.0 | (7.2;8.8) | 9.7 | (8.9;10.6) | 9.1 | (8.3;9.9) | 9.2 | (8.4;10.0) | 9.5 | (8.7;10.4) | 10.7 | (9.8;11.6) | 1.0 | | (-1.1 | ; | 3.1) |
| 19-29 | 1.4 | (0.2;2.5) | 0.9 | (0.0;1.7) | 1.7 | (0.6;2.8) | 0.3 | (;0.1;0.8) | 0.6 | (0.0;1.2) | 0.6 | (0.0;1.2) | 1.5 | (0.3;2.7) | 0.8 | (;0.2;1.7) | 1.8 | (0.4;3.1) | 0.7 | (0.2;1.2) | -1.2 | | (-13.6 | ; | 12.9) |
| 30-39 | 2.9 | (1.6;4.2) | 3.1 | (1.8;4.4) | 2.7 | (1.5;3.9) | 2.5 | (1.3;3.8) | 3.1 | (1.7;4.4) | 3.0 | (1.4;4.5) | 2.3 | (1.2;3.4) | 3.2 | (1.8;4.5) | 2.7 | (1.6;3.9) | 4.4 | (2.8;6.0) | 2.8 | | (-1.5 | ; | 7.3) |
| 40-49 | 8.4 | (6.1;10.7) | 5.8 | (3.8;7.8) | 8.3 | (6.6;10.0) | 9.5 | (7.0;12.0) | 7.1 | (5.2;9.1) | 8.7 | (6.7;10.7) | 7.3 | (5.4;9.2) | 8.3 | (6.2;10.5) | 8.1 | (5.8;10.4) | 9.0 | (6.7;11.2) | 0.9 | | (-2.3 | ; | 4.3) |
| 50-59 | 15.6 | (13.2;18.0) | 14.2 | (11.6;16.8) | 15.6 | (12.8;18.4) | 14.1 | (11.5;16.7) | 10.3 | (8.2;12.5) | 16.0 | (13.5;18.6) | 16.4 | (13.9;18.8) | 14.6 | (12.1;17.2) | 14.4 | (12.2;16.5) | 19.1 | (16.2;21.9) | 1.6 | | (-1.9 | ; | 5.1) |
| 60-69 | 23.2 | (20.1;26.3) | 24.6 | (21.5;27.6) | 31.1 | (27.2;34.9) | 25.6 | (22.0;29.3) | 21.5 | (18.3;24.8) | 24.2 | (21.0;27.4) | 21.0 | (18.0;24.0) | 22.3 | (19.5;25.1) | 24.2 | (21.3;27.1) | 25.1 | (22.0;28.2) | -1.0 | | (-3.8 | ; | 1.8) |
| 70+ | 25.9 | (22.2;29.6) | 26.5 | (23.1;29.8) | 34.8 | (29.4;40.2) | 27.0 | (23.1;30.9) | 27.1 | (23.7;30.5) | 31.8 | (28.2;35.4) | 30.4 | (26.9;34.0) | 29.2 | (25.9;32.4) | 31.0 | (27.8;34.2) | 31.5 | (28.4;34.6) | 1.6 | | (-0.3 | ; | 3.6) |
|  |  |  |  |  |  |  |  |  |  |  |  |  |  |  |  |  |  |  |  |  |  | |  |  |  |
| Men, age≥19 | 11.1 | (9.8;12.4) | 9.4 | (8.2;10.6) | 12.0 | (10.6;13.4) | 11.2 | (9.8;12.7) | 9.1 | (7.8;10.3) | 11.1 | (9.9;12.4) | 10.9 | (9.7;12.0) | 11.2 | (9.8;12.6) | 11.1 | (9.9;12.3) | 13.0 | (11.5;14.5) | 1.5 | | (-1.0 | ; | 4.0) |
| 19-29 | 1.9 | (;0.1;3.9) | 0.9 | (;0.4;2.3) | 1.9 | (0.2;3.6) | 0.5 | (;0.2;1.1) | 0.7 | (;0.3;1.6) | 0.5 | (;0.2;1.1) | 2.3 | (0.2;4.4) | 1.2 | (;0.5;2.9) | 1.1 | (;0.4;2.5) | 0.6 | (0.0;1.2) | -6.3 | | (-19.5 | ; | 9.2) |
| 30-39 | 4.2 | (1.9;6.4) | 3.3 | (1.2;5.3) | 3.9 | (1.7;6.2) | 2.2 | (0.6;3.9) | 2.8 | (0.8;4.7) | 4.0 | (1.4;6.7) | 3.5 | (1.5;5.5) | 3.7 | (1.6;5.7) | 3.1 | (1.3;4.9) | 5.9 | (3.3;8.5) | 3.0 | | (-3.1 | ; | 9.4) |
| 40-49 | 11.0 | (7.3;14.8) | 5.3 | (2.9;7.7) | 11.4 | (8.1;14.6) | 12.9 | (8.8;17.0) | 9.7 | (6.3;13.1) | 10.9 | (7.9;13.9) | 9.3 | (6.5;12.1) | 13.0 | (8.8;17.2) | 11.5 | (8.0;15.0) | 14.1 | (10.2;18.0) | 3.4 | | (-2.0 | ; | 9.2) |
| 50-59 | 20.7 | (16.4;25.1) | 17.0 | (12.7;21.3) | 20.0 | (15.4;24.7) | 17.5 | (13.2;21.8) | 12.9 | (9.5;16.3) | 19.6 | (15.6;23.5) | 20.6 | (17.3;23.9) | 17.9 | (14.2;21.6) | 18.9 | (14.9;22.9) | 23.8 | (19.4;28.3) | 1.5 | | (-2.3 | ; | 5.5) |
| 60-69 | 26.3 | (22.6;30.0) | 29.2 | (24.4;34.1) | 33.6 | (27.6;39.5) | 32.6 | (27.6;37.6) | 24.4 | (19.5;29.2) | 27.9 | (22.5;33.3) | 23.7 | (19.0;28.4) | 25.7 | (21.6;29.8) | 27.2 | (22.7;31.7) | 28.8 | (24.1;33.6) | -0.9 | | (-3.5 | ; | 1.9) |
| 70+ | 23.6 | (17.8;29.3) | 26.5 | (21.5;31.6) | 27.1 | (20.3;33.9) | 27.3 | (21.3;33.2) | 25.4 | (20.3;30.5) | 29.1 | (24.1;34.2) | 30.2 | (25.0;35.3) | 28.8 | (23.2;34.5) | 30.0 | (24.9;35.2) | 28.7 | (24.1;33.4) | 1.9* | | (0.6 | ; | 3.2) |
|  |  |  |  |  |  |  |  |  |  |  |  |  |  |  |  |  |  |  |  |  |  | |  |  |  |
| Women, age≥19 | 7.3 | (6.4;8.2) | 7.8 | (6.8;8.9) | 9.2 | (8.1;10.4) | 7.5 | (6.6;8.4) | 7.0 | (6.0;8.0) | 8.3 | (7.3;9.2) | 7.3 | (6.3;8.2) | 7.1 | (6.3;8.0) | 8.0 | (7.0;8.9) | 8.2 | (7.3;9.1) | -0.1 | | (-2.3 | ; | 2.2) |
| 19-29 | 0.8 | (;0.2;1.8) | 0.8 | (;0.3;1.9) | 1.4 | (0.2;2.7) | 0.2 | (;0.2;0.7) | 0.5 | (;0.2;1.2) | 0.8 | (;0.4;1.9) | 0.6 | (;0.2;1.4) | 0.3 | (;0.2;0.9) | 2.5 | (0.2;4.9) | 0.8 | (;0.1;1.7) | 3.1 | | (-12.2 | ; | 21.0) |
| 30-39 | 1.6 | (0.6;2.6) | 2.9 | (1.2;4.7) | 1.4 | (0.4;2.4) | 2.8 | (1.2;4.5) | 3.4 | (1.4;5.4) | 1.8 | (0.6;3.0) | 0.9 | (0.2;1.7) | 2.7 | (1.0;4.3) | 2.3 | (0.7;3.9) | 2.7 | (1.1;4.3) | 2.0 | | (-7.2 | ; | 12.2) |
| 40-49 | 5.7 | (3.4;8.0) | 6.3 | (3.7;8.9) | 5.2 | (3.4;7.0) | 6.2 | (3.9;8.5) | 4.6 | (2.6;6.6) | 6.4 | (3.9;8.9) | 5.2 | (3.0;7.5) | 3.5 | (2.2;4.9) | 4.7 | (2.5;6.8) | 3.7 | (1.8;5.5) | -4.7 | | (-8.7 | ; | -0.4) |
| 50-59 | 10.6 | (7.9;13.2) | 11.4 | (8.5;14.3) | 11.5 | (8.6;14.4) | 10.8 | (7.9;13.7) | 7.8 | (5.2;10.4) | 12.4 | (9.5;15.3) | 12.1 | (8.8;15.3) | 11.3 | (8.4;14.2) | 9.7 | (7.0;12.5) | 14.3 | (10.6;18.1) | 1.4 | | (-2.2 | ; | 5.2) |
| 60-69 | 20.4 | (16.2;24.5) | 20.3 | (16.4;24.2) | 28.7 | (23.7;33.7) | 19.5 | (14.9;24.1) | 18.9 | (14.5;23.4) | 20.6 | (16.7;24.6) | 18.4 | (14.4;22.4) | 19.0 | (14.8;23.3) | 21.4 | (17.8;25.1) | 21.6 | (18.0;25.3) | -0.9 | | (-4.2 | ; | 2.5) |
| 70+ | 27.5 | (22.6;32.4) | 26.4 | (21.8;31.0) | 40.4 | (33.2;47.6) | 26.8 | (22.0;31.6) | 28.3 | (23.2;33.5) | 33.6 | (28.7;38.5) | 30.6 | (25.9;35.3) | 29.4 | (25.3;33.6) | 31.6 | (27.6;35.6) | 33.5 | (29.7;37.3) | 1.3 | | (-1.6 | ; | 4.3) |
| Household income | |  |  |  |  |  |  |  |  |  |  |  |  |  |  |  |  |  |  |  |  | |  |  |  |
| Low | 9.6 | (7.8;11.4) | 9.3 | (7.8;10.9) | 12.4 | (10.4;14.4) | 12.7 | (10.3;15.2) | 10.6 | (8.7;12.5) | 12.0 | (10.0;14.0) | 12.2 | (10.3;14.1) | 11.5 | (9.7;13.2) | 10.6 | (8.5;12.8) | 14.8 | (12.2;17.3) | 2.8 | | (-0.2 | ; | 6.0) |
| Low-middle | 8.1 | (6.5;9.7) | 10.6 | (8.6;12.7) | 11.2 | (9.5;12.9) | 9.5 | (7.7;11.3) | 9.6 | (7.8;11.4) | 9.4 | (7.6;11.2) | 9.1 | (7.6;10.6) | 11.3 | (9.2;13.5) | 10.4 | (8.6;12.2) | 11.4 | (9.7;13.1) | 1.6 | | (-1.1 | ; | 4.3) |
| Middle | 10.5 | (8.4;12.6) | 6.4 | (4.9;7.9) | 10.3 | (8.6;12.0) | 7.8 | (5.9;9.6) | 6.4 | (4.8;8.0) | 10.2 | (8.4;11.9) | 9.2 | (7.6;10.8) | 8.2 | (6.5;9.9) | 9.2 | (7.5;10.8) | 10.6 | (8.6;12.6) | 1.0 | | (-3.6 | ; | 5.7) |
| Middle-high | 7.9 | (6.3;9.4) | 8.6 | (6.5;10.7) | 9.9 | (8.1;11.8) | 7.9 | (6.3;9.5) | 6.0 | (4.6;7.3) | 8.4 | (6.9;9.9) | 8.2 | (6.6;9.8) | 8.2 | (6.5;9.9) | 8.7 | (7.0;10.3) | 8.9 | (7.4;10.4) | 0.5 | | (-2.6 | ; | 3.6) |
| High | 10.3 | (8.2;12.3) | 7.6 | (5.6;9.6) | 9.7 | (7.9;11.5) | 8.7 | (6.7;10.7) | 7.8 | (6.2;9.4) | 8.4 | (6.8;10.0) | 7.4 | (5.9;8.8) | 6.7 | (5.4;8.0) | 8.9 | (7.4;10.4) | 8.1 | (6.7;9.6) | -2.1 | | (-4.9 | ; | 0.7) |
| Values are presented as weighted % (95% confidence interval). Age-standardized prevalence was calculated using the 2005 Population Projections for Korea.  *The annual percent change (APC) is significantly different from 0. | | | | | | | | | | | | | | | | | | | | | | | | | |
|  |  |  |  |  |  |  |  |  |  |  |  |  |  |  |  |  |  |  |  |  |  |  | |  |  |
