# Supplementary material for: Obesity, hypertension, diabetes mellitus, and hypercholesterolemia in Korean adults before and during the COVID-19 pandemic: a special report of the 2020 Korea National Health and Nutrition Examination Survey
Source: Epidemiol Health. 2022 Apr 25;44:e2022041. doi: 10.4178/epih.e2022041 (PMC9133598; doi:10.4178/epih.e2022041)
Supplement: Supplementary Material 4 — Prevalence of hypercholesterolemia by sex and age using the Korea National Health and Nutrition Examination Survey (KNHANES) from 2011 to 20201 [file epih-44-e2022041-suppl4.docx]

| Supplementary Material 4. Prevalence of hypercholesterolemia by sex and age using the Korea National Health and Nutrition Examination Survey (KNHANES) from 2011 to 2020^1^ | | | | | | | | | | | | | | | | | | | | | | | | | |
| --- | --- | --- | --- | --- | --- | --- | --- | --- | --- | --- | --- | --- | --- | --- | --- | --- | --- | --- | --- | --- | --- | --- | --- | --- | --- |
| Characteristics | 2011 | | 2012 | | 2013 | | 2014 | | 2015 | | 2016 | | 2017 | | 2018 | | 2019 | | 2020 | | Annual Percent Change | | | | |
| Total, age≥19 | 11.4 | (10.4;12.3) | 11.9 | (10.9;13.0) | 12.3 | (11.4;13.2) | 11.6 | (10.5;12.6) | 14.8 | (13.6;15.9) | 16.7 | (15.5;17.9) | 18.1 | (16.9;19.3) | 17.7 | (16.4;19.0) | 18.1 | (17.0;19.2) | 19.7 | (18.5;20.9) | 6.9* | (5.0 | ; | 8.8) |  |
| 19-29 | 3.0 | (1.5;4.5) | 3.4 | (1.8;4.9) | 3.4 | (1.9;4.9) | 1.3 | (0.3;2.4) | 4.1 | (2.4;5.8) | 5.5 | (2.9;8.2) | 6.3 | (4.0;8.6) | 5.2 | (3.2;7.2) | 3.9 | (2.1;5.6) | 5.2 | (3.3;7.0) | 6.9 | (-0.6 | ; | 15.0) |  |
| 30-39 | 7.7 | (5.9;9.5) | 8.6 | (6.4;10.8) | 8.5 | (6.5;10.4) | 6.0 | (4.2;7.8) | 8.2 | (5.9;10.6) | 9.4 | (7.3;11.6) | 11.5 | (9.0;13.9) | 12.3 | (9.7;14.9) | 10.0 | (7.6;12.3) | 10.6 | (8.3;12.9) | 4.8* | (1.0 | ; | 8.8) |  |
| 40-49 | 10.0 | (8.0;12.0) | 9.2 | (7.0;11.5) | 10.1 | (8.1;12.1) | 12.6 | (9.6;15.6) | 15.6 | (13.1;18.0) | 15.0 | (12.8;17.1) | 18.1 | (15.0;21.3) | 16.8 | (13.7;19.8) | 16.4 | (14.0;18.8) | 21.2 | (18.0;24.3) | 8.6* | (5.5 | ; | 11.7) |  |
| 50-59 | 19.3 | (17.0;21.7) | 22.0 | (18.9;25.1) | 20.3 | (17.6;23.0) | 18.9 | (15.6;22.3) | 22.4 | (19.3;25.5) | 30.9 | (27.6;34.1) | 28.2 | (25.4;31.1) | 26.3 | (23.3;29.4) | 31.2 | (28.1;34.2) | 29.4 | (26.2;32.7) | 5.5* | (2.6 | ; | 8.4) |  |
| 60-69 | 26.8 | (23.5;30.1) | 24.2 | (20.3;28.1) | 26.9 | (23.0;30.8) | 26.9 | (23.2;30.7) | 32.2 | (29.0;35.4) | 37.6 | (33.7;41.4) | 33.7 | (30.5;37.0) | 35.9 | (32.4;39.3) | 42.8 | (39.3;46.3) | 41.9 | (37.8;45.9) | 6.3* | (4.2 | ; | 8.4) |  |
| 70+ | 17.3 | (14.8;19.9) | 21.7 | (18.4;24.9) | 24.0 | (20.0;27.9) | 23.2 | (19.4;27.0) | 28.4 | (24.2;32.5) | 23.9 | (20.6;27.2) | 35.4 | (31.6;39.2) | 35.9 | (32.3;39.5) | 35.1 | (31.4;38.9) | 40.4 | (36.6;44.1) | 9.0* | (6.4 | ; | 11.6) |  |
|  |  |  |  |  |  |  |  |  |  |  |  |  |  |  |  |  |  |  |  |  |  |  |  |  |  |
| Men, age≥19 | 10.6 | (8.9;12.2) | 10.3 | (8.8;11.8) | 11.6 | (10.3;12.9) | 11.0 | (9.5;12.5) | 14.1 | (12.4;15.8) | 16.5 | (14.8;18.2) | 17.5 | (15.7;19.3) | 17.9 | (15.8;19.9) | 17.0 | (15.3;18.6) | 20.2 | (18.5;22.0) | 8.0* | (5.7 | ; | 10.3) |  |
| 19-29 | 3.8 | (1.3;6.3) | 3.7 | (1.1;6.2) | 4.9 | (2.3;7.4) | 1.1 | (;0.3;2.5) | 6.3 | (3.2;9.4) | 6.8 | (2.9;10.6) | 8.7 | (4.9;12.5) | 7.3 | (3.9;10.8) | 3.3 | (1.2;5.4) | 5.3 | (2.9;7.8) | 4.4 | (-6.0 | ; | 16.0) |  |
| 30-39 | 10.3 | (6.9;13.7) | 11.3 | (7.6;14.9) | 11.9 | (8.4;15.3) | 7.6 | (4.4;10.7) | 10.6 | (6.7;14.4) | 10.8 | (7.7;13.8) | 14.8 | (11.1;18.4) | 15.1 | (11.0;19.2) | 12.5 | (8.7;16.4) | 12.7 | (8.8;16.6) | 3.5 | (-0.7 | ; | 7.9) |  |
| 40-49 | 10.4 | (7.5;13.2) | 8.1 | (5.3;10.9) | 12.1 | (8.8;15.3) | 16.3 | (11.3;21.3) | 18.6 | (14.6;22.6) | 20.2 | (16.5;24.0) | 18.3 | (14.0;22.7) | 22.9 | (18.0;27.8) | 20.4 | (16.6;24.2) | 28.2 | (22.9;33.5) | 11.1* | (6.7 | ; | 15.6) |  |
| 50-59 | 16.2 | (12.2;20.3) | 16.0 | (11.8;20.3) | 13.5 | (9.8;17.2) | 18.3 | (13.3;23.3) | 16.2 | (12.7;19.8) | 28.1 | (23.0;33.3) | 24.8 | (20.9;28.6) | 20.8 | (17.0;24.7) | 26.0 | (21.8;30.2) | 27.2 | (22.8;31.5) | 7.0* | (2.6 | ; | 11.6) |  |
| 60-69 | 17.0 | (13.1;20.8) | 16.6 | (11.7;21.4) | 18.8 | (14.1;23.5) | 20.3 | (15.7;24.9) | 23.2 | (18.9;27.4) | 27.2 | (22.1;32.4) | 25.8 | (21.3;30.3) | 27.0 | (22.7;31.2) | 32.2 | (27.0;37.4) | 35.4 | (30.2;40.5) | 8.7* | (7.1 | ; | 10.3) |  |
| 70+ | 13.5 | (9.7;17.4) | 14.5 | (9.9;19.0) | 16.8 | (11.0;22.7) | 9.7 | (6.1;13.4) | 20.1 | (14.7;25.4) | 16.4 | (12.2;20.7) | 25.3 | (20.9;29.7) | 25.7 | (21.4;30.0) | 25.0 | (20.6;29.4) | 32.4 | (26.4;38.4) | 10.5* | (5.8 | ; | 15.4) |  |
|  |  |  |  |  |  |  |  |  |  |  |  |  |  |  |  |  |  |  |  |  |  |  |  |  |  |
| Women, age≥19 | 12.0 | (10.8;13.1) | 13.3 | (11.9;14.8) | 12.6 | (11.4;13.9) | 11.9 | (10.7;13.1) | 15.1 | (13.8;16.4) | 16.5 | (15.2;17.8) | 18.3 | (17.0;19.6) | 17.1 | (15.8;18.5) | 18.9 | (17.5;20.3) | 18.8 | (17.3;20.3) | 5.9* | (3.9 | ; | 8.0) |  |
| 19-29 | 2.2 | (0.6;3.8) | 3.0 | (1.1;5.0) | 1.7 | (0.5;2.8) | 1.6 | (0.0;3.1) | 1.6 | (0.1;3.1) | 4.1 | (1.7;6.6) | 3.6 | (1.6;5.5) | 2.7 | (0.9;4.5) | 4.5 | (1.8;7.1) | 5.0 | (2.7;7.4) | 10.0* | (2.5 | ; | 18.1) |  |
| 30-39 | 5.0 | (2.9;7.1) | 5.7 | (3.2;8.3) | 4.9 | (3.0;6.8) | 4.4 | (2.4;6.4) | 5.8 | (3.3;8.2) | 8.0 | (5.3;10.7) | 7.7 | (4.9;10.6) | 9.2 | (6.5;11.9) | 7.2 | (4.8;9.7) | 8.2 | (5.4;11.1) | 7.1* | (2.8 | ; | 11.6) |  |
| 40-49 | 9.6 | (6.5;12.7) | 10.4 | (6.8;13.9) | 8.0 | (5.7;10.4) | 9.1 | (6.0;12.2) | 12.6 | (9.3;16.0) | 9.6 | (6.9;12.3) | 17.9 | (14.1;21.8) | 10.5 | (7.9;13.1) | 12.3 | (9.5;15.1) | 13.8 | (10.1;17.6) | 4.8 | (-1.3 | ; | 11.2) |  |
| 50-59 | 22.4 | (19.0;25.8) | 28.0 | (23.5;32.4) | 26.8 | (22.8;30.9) | 19.6 | (15.6;23.5) | 28.5 | (24.4;32.7) | 33.7 | (29.6;37.8) | 31.7 | (27.5;35.9) | 31.9 | (27.8;36.0) | 36.4 | (31.7;41.1) | 31.7 | (27.5;35.9) | 4.3* | (1.3 | ; | 7.5) |  |
| 60-69 | 35.8 | (31.0;40.5) | 31.2 | (25.3;37.1) | 34.5 | (29.0;39.9) | 32.8 | (27.1;38.5) | 40.6 | (35.6;45.6) | 47.3 | (41.7;53.0) | 41.4 | (36.9;46.0) | 44.3 | (39.3;49.4) | 52.5 | (47.7;57.3) | 48.0 | (42.9;53.0) | 4.9* | (2.6 | ; | 7.4) |  |
| 70+ | 19.8 | (16.2;23.5) | 26.4 | (22.0;30.8) | 29.0 | (23.6;34.5) | 34.0 | (28.2;39.8) | 34.4 | (29.1;39.7) | 29.0 | (24.2;33.9) | 42.6 | (37.6;47.6) | 42.9 | (38.0;47.8) | 42.2 | (36.9;47.5) | 46.0 | (40.7;51.3) | 8.1* | (5.1 | ; | 11.1) |  |
| Household income | |  |  |  |  |  |  |  |  |  |  |  |  |  |  |  |  |  |  |  |  |  |  |  |  |
| Low | 12.1 | (10.1;14.1) | 11.0 | (8.9;13.0) | 12.5 | (10.1;15.0) | 10.8 | (8.8;12.9) | 15.5 | (12.8;18.1) | 17.2 | (14.7;19.6) | 19.8 | (17.3;22.2) | 17.0 | (14.6;19.3) | 16.8 | (14.5;19.1) | 19.9 | (17.0;22.8) | 6.5* | (3.0 | ; | 10.0) |  |
| Low-middle | 11.3 | (9.3;13.3) | 14.1 | (11.6;16.6) | 10.6 | (8.7;12.5) | 11.7 | (9.1;14.3) | 13.5 | (11.2;15.8) | 13.9 | (11.9;16.0) | 16.9 | (14.6;19.2) | 19.6 | (16.7;22.5) | 18.9 | (16.5;21.3) | 19.5 | (16.9;22.1) | 7.0* | (4.2 | ; | 9.8) |  |
| Middle | 10.7 | (8.8;12.7) | 9.7 | (7.4;12.1) | 12.1 | (10.1;14.0) | 12.5 | (10.0;14.9) | 14.1 | (11.8;16.4) | 17.6 | (14.8;20.3) | 18.2 | (15.6;20.8) | 18.3 | (15.7;20.9) | 18.2 | (15.6;20.8) | 18.9 | (16.4;21.4) | 7.5* | (5.1 | ; | 10.0) |  |
| Middle-high | 10.6 | (8.7;12.5) | 12.0 | (9.7;14.2) | 13.4 | (11.0;15.8) | 10.9 | (8.6;13.1) | 15.2 | (12.4;18.0) | 17.5 | (14.5;20.5) | 16.1 | (13.6;18.6) | 16.7 | (14.0;19.5) | 18.8 | (16.5;21.1) | 18.8 | (16.4;21.3) | 6.5* | (4.3 | ; | 8.7) |  |
| High | 12.1 | (10.0;14.3) | 13.5 | (11.0;16.0) | 13.3 | (10.8;15.8) | 12.2 | (10.0;14.4) | 15.2 | (12.6;17.7) | 16.8 | (14.4;19.1) | 19.5 | (16.9;22.1) | 16.7 | (14.3;19.2) | 17.8 | (15.7;19.9) | 21.5 | (18.7;24.3) | 6.1* | (3.6 | ; | 8.6) |  |
| Values are presented as weighted % (95% confidence interval). Age-standardized prevalence was calculated using the 2005 Population Projections for Korea.  *The annual percent change (APC) is significantly different from 0. | | | | | | | | | | | | | | | | | | | | | | | | | |
