# Supplementary material for: Obesity, hypertension, diabetes mellitus, and hypercholesterolemia in Korean adults before and during the COVID-19 pandemic: a special report of the 2020 Korea National Health and Nutrition Examination Survey
Source: Epidemiol Health. 2022 Apr 25;44:e2022041. doi: 10.4178/epih.e2022041 (PMC9133598; doi:10.4178/epih.e2022041)
Supplement: Supplementary Material 5 — Prevalence of severe obesity (body mass index≥30kg/m2) by sex and age using the Korea National Health and Nutrition Examination Survey (KNHANES) from 2011 to 20201 [file epih-44-e2022041-suppl5.docx]

| Supplementary Material 5. Prevalence of severe obesity (body mass index≥30kg/m^2^) by sex and age using the Korea National Health and Nutrition Examination Survey (KNHANES) from 2011 to 2020^1^ | | | | | | | | | | | | | | | | | | | | | | | | | | |
| --- | --- | --- | --- | --- | --- | --- | --- | --- | --- | --- | --- | --- | --- | --- | --- | --- | --- | --- | --- | --- | --- | --- | --- | --- | --- | --- |
| Characteristics | 2011 | | 2012 | | 2013 | | 2014 | | 2015 | | 2016 | | 2017 | | 2018 | | 2019 | | 2020 | | Annual Percent Change | | | | |  |
| Total, age≥19 | 4.5 | (3.7;5.3) | 5.0 | (4.1;6.0) | 4.8 | (4.1;5.5) | 4.5 | (3.7;5.3) | 5.8 | (4.9;6.6) | 5.7 | (5.0;6.5) | 6.1 | (5.2;7.0) | 6.4 | (5.3;7.4) | 6.2 | (5.4;7.0) | 8.2 | (7.1;9.2) | 6.1* | (3.8 | ; | 8.4) |  |  |
| 19-29 | 6.1 | (3.5;8.7) | 6.2 | (3.7;8.7) | 4.6 | (3.1;6.2) | 4.3 | (2.6;6.1) | 5.4 | (3.4;7.4) | 5.7 | (3.8;7.7) | 7.7 | (5.4;10.1) | 7.3 | (5.1;9.6) | 7.5 | (5.4;9.6) | 10.9 | (8.3;13.5) | 8.4 * | (3.3 | ; | 13.7) |  |  |
| 30-39 | 5.1 | (3.3;6.9) | 6.0 | (4.1;7.9) | 5.3 | (3.9;6.8) | 6.3 | (4.5;8.1) | 9.4 | (7.0;11.8) | 6.3 | (4.9;7.6) | 7.3 | (5.3;9.3) | 9.2 | (6.4;11.9) | 6.7 | (4.9;8.4) | 10.2 | (7.7;12.7) | 6.0 * | (0.9 | ; | 11.4) |  |  |
| 40-49 | 4.7 | (3.3;6.1) | 4.9 | (3.3;6.5) | 5.0 | (3.5;6.5) | 4.1 | (2.8;5.4) | 4.9 | (3.2;6.7) | 6.7 | (5.1;8.3) | 6.6 | (4.9;8.3) | 5.6 | (3.9;7.2) | 6.9 | (5.4;8.4) | 8.2 | (6.3;10.1) | 6.2 * | (2.9 | ; | 9.5) |  |  |
| 50-59 | 2.7 | (1.7;3.7) | 3.4 | (2.2;4.6) | 4.0 | (2.5;5.4) | 3.7 | (2.5;4.9) | 3.6 | (2.5;4.6) | 4.9 | (3.5;6.3) | 2.9 | (1.9;3.9) | 3.6 | (2.4;4.8) | 5.2 | (3.9;6.4) | 5.2 | (3.6;6.8) | 5.5 * | (0.6 | ; | 10.5) |  |  |
| 60-69 | 2.8 | (1.6;4.0) | 3.6 | (2.5;4.8) | 5.9 | (3.9;7.9) | 3.8 | (2.5;5.1) | 3.6 | (2.4;4.8) | 4.9 | (3.4;6.5) | 4.1 | (2.9;5.3) | 4.6 | (3.1;6.1) | 4.4 | (3.0;5.8) | 4.5 | (3.0;6.0) | 2.4 | (-2.5 | ; | 7.6) |  |  |
| 70+ | 2.8 | (1.6;3.9) | 3.9 | (2.5;5.3) | 3.5 | (2.0;5.0) | 3.1 | (2.1;4.0) | 4.6 | (3.2;6.0) | 3.7 | (2.4;5.0) | 3.7 | (2.5;4.9) | 4.6 | (3.0;6.1) | 2.8 | (1.7;3.9) | 3.7 | (2.5;4.9) | 1.1 | (-3.6 | ; | 6.1) |  |  |
|  |  |  |  |  |  |  |  |  |  |  |  |  |  |  |  |  |  |  |  |  |  |  |  |  |  |  |
| Men, age≥19 | 4.2 | (3.1;5.2) | 5.1 | (3.7;6.5) | 5.3 | (4.3;6.3) | 5.0 | (3.8;6.2) | 6.8 | (5.4;8.1) | 6.3 | (5.2;7.5) | 6.3 | (5.1;7.6) | 7.6 | (6.1;9.1) | 6.7 | (5.6;7.9) | 10.0 | (8.5;11.6) | 7.9* | (4.6 | ; | 11.2) |  |  |
| 19-29 | 6.9 | (3.3;10.5) | 9.6 | (5.1;14.2) | 7.1 | (4.6;9.7) | 5.3 | (2.5;8.1) | 7.4 | (4.1;10.7) | 7.7 | (4.6;10.8) | 9.3 | (6.0;12.7) | 11.2 | (7.5;14.9) | 9.7 | (6.3;13.1) | 14.3 | (10.6;18.0) | 8.1 * | (3.0 | ; | 13.3) |  |  |
| 30-39 | 5.2 | (2.7;7.8) | 6.7 | (3.8;9.6) | 6.1 | (3.8;8.5) | 8.1 | (5.2;11.0) | 12.8 | (8.8;16.9) | 8.2 | (5.9;10.5) | 9.9 | (6.6;13.1) | 11.1 | (7.3;15.0) | 7.5 | (4.6;10.5) | 13.8 | (9.8;17.9) | 8.0 * | (1.3 | ; | 15.1) |  |  |
| 40-49 | 4.8 | (2.7;6.8) | 4.2 | (2.0;6.4) | 6.6 | (4.2;9.0) | 4.9 | (2.8;7.0) | 6.1 | (3.5;8.7) | 7.6 | (5.3;9.8) | 5.7 | (3.5;7.9) | 7.0 | (4.5;9.5) | 7.3 | (5.0;9.7) | 10.2 | (7.1;13.3) | 7.1 * | (2.7 | ; | 11.7) |  |  |
| 50-59 | 0.6 | (;0.2;1.4) | 1.5 | (0.4;2.5) | 3.5 | (1.6;5.5) | 2.8 | (1.2;4.4) | 2.4 | (1.2;3.5) | 4.2 | (2.1;6.2) | 2.0 | (0.8;3.1) | 2.5 | (1.0;4.0) | 5.3 | (3.2;7.4) | 4.9 | (2.9;6.8) | 12.3* | (1.7 | ; | 23.9) |  |  |
| 60-69 | 2.2 | (0.6;3.7) | 0.7 | (0.1;1.4) | 2.6 | (0.4;4.8) | 3.0 | (1.1;4.8) | 1.6 | (0.5;2.7) | 2.9 | (1.3;4.5) | 2.5 | (0.9;4.1) | 3.3 | (1.3;5.3) | 2.4 | (1.1;3.7) | 3.7 | (1.7;5.7) | 7.8 | (-1.7 | ; | 18.3) |  |  |
| 70+ | 0.1 | (;0.1;0.4) | 1.6 | (0.0;3.1) | 0.2 | (;0.2;0.5) | 1.5 | (0.5;2.6) | 2.6 | (0.8;4.4) | 1.3 | (0.2;2.5) | 1.1 | (0.2;1.9) | 2.2 | (1.0;3.5) | 1.4 | (0.2;2.6) | 2.3 | (0.8;3.9) | 9.1 | (-7.5 | ; | 28.7) |  |  |
|  |  |  |  |  |  |  |  |  |  |  |  |  |  |  |  |  |  |  |  |  |  |  |  |  |  |  |
| Women, age≥19 | 4.7 | (3.6;5.8) | 4.8 | (3.8;5.9) | 4.1 | (3.4;4.9) | 3.9 | (3.0;4.7) | 4.5 | (3.7;5.4) | 4.9 | (4.0;5.8) | 5.6 | (4.5;6.6) | 4.9 | (4.0;5.9) | 5.5 | (4.5;6.6) | 6.1 | (4.8;7.3) | 3.7* | (1.0 | ; | 6.5) |  |  |
| 19-29 | 5.2 | (2.3;8.0) | 2.4 | (1.0;3.9) | 1.8 | (0.5;3.0) | 3.3 | (1.4;5.2) | 3.2 | (1.1;5.2) | 3.5 | (1.6;5.4) | 5.9 | (3.0;8.8) | 2.9 | (1.2;4.5) | 5.0 | (2.3;7.6) | 7.2 | (3.9;10.5) | 7.5 | (-2.2 | ; | 18.2) |  |  |
| 30-39 | 4.9 | (2.7;7.1) | 5.3 | (2.9;7.6) | 4.4 | (2.5;6.3) | 4.3 | (2.3;6.3) | 5.7 | (3.5;7.9) | 4.1 | (2.3;5.9) | 4.5 | (2.4;6.5) | 7.0 | (4.3;9.7) | 5.7 | (3.6;7.7) | 6.1 | (3.5;8.7) | 3.0 | (-1.1 | ; | 7.2) |  |  |
| 40-49 | 4.6 | (2.5;6.6) | 5.6 | (3.2;8.1) | 3.3 | (1.6;5.0) | 3.2 | (1.6;4.7) | 3.8 | (1.9;5.6) | 5.8 | (3.6;7.9) | 7.5 | (5.0;10.0) | 4.1 | (2.5;5.8) | 6.5 | (4.2;8.7) | 6.1 | (4.1;8.1) | 4.5 | (-2.2 | ; | 11.5) |  |  |
| 50-59 | 4.8 | (3.0;6.6) | 5.3 | (3.2;7.4) | 4.5 | (2.5;6.4) | 4.6 | (2.7;6.4) | 4.8 | (2.8;6.7) | 5.7 | (3.6;7.7) | 3.8 | (2.3;5.4) | 4.7 | (3.0;6.4) | 5.1 | (3.4;6.7) | 5.5 | (3.2;7.8) | 0.5 | (-2.5 | ; | 3.6) |  |  |
| 60-69 | 3.4 | (1.8;4.9) | 6.3 | (4.1;8.5) | 8.8 | (5.6;12.1) | 4.6 | (2.8;6.4) | 5.5 | (3.5;7.5) | 6.8 | (4.4;9.2) | 5.7 | (3.9;7.4) | 5.8 | (3.5;8.2) | 6.3 | (3.9;8.7) | 5.3 | (3.1;7.5) | 0.8 | (-5.5 | ; | 7.4) |  |  |
| 70+ | 4.4 | (2.5;6.3) | 5.4 | (3.2;7.6) | 5.6 | (3.2;8.1) | 4.1 | (2.5;5.6) | 5.8 | (3.9;7.8) | 5.3 | (3.3;7.3) | 5.5 | (3.5;7.5) | 6.2 | (3.7;8.7) | 3.7 | (2.0;5.4) | 4.6 | (2.9;6.3) | -0.4 | (-4.7 | ; | 4.2) |  |  |
| Household income | |  |  |  |  |  |  |  |  |  |  |  |  |  |  |  |  |  |  |  |  |  |  |  |  |  |
| Low | 6.3 | (4.2;8.5) | 8.5 | (6.1;10.9) | 7.4 | (5.6;9.2) | 6.0 | (4.4;7.7) | 9.1 | (6.7;11.4) | 7.9 | (6.1;9.7) | 8.3 | (6.3;10.4) | 8.4 | (6.4;10.4) | 8.5 | (6.3;10.6) | 10.0 | (7.5;12.5) | 3.3* | (0.0 | ; | 6.7) |  |  |
| Low-middle | 4.2 | (2.5;6.0) | 5.9 | (3.9;7.9) | 5.1 | (3.6;6.6) | 4.7 | (3.1;6.2) | 6.3 | (4.4;8.2) | 5.3 | (3.8;6.8) | 5.9 | (4.3;7.4) | 7.1 | (4.9;9.2) | 6.7 | (4.6;8.7) | 8.2 | (6.2;10.3) | 5.7* | (2.6 | ; | 9.0) |  |  |
| Middle | 5.6 | (3.6;7.5) | 2.8 | (1.3;4.3) | 3.9 | (2.6;5.3) | 3.8 | (2.4;5.2) | 4.1 | (2.6;5.6) | 6.2 | (4.6;7.8) | 6.6 | (4.3;8.8) | 6.9 | (4.9;9.0) | 5.8 | (4.3;7.3) | 8.8 | (6.3;11.3) | 7.9* | (1.8 | ; | 14.4) |  |  |
| Middle-high | 2.8 | (1.6;4.0) | 3.8 | (2.3;5.2) | 4.1 | (2.9;5.3) | 4.1 | (2.6;5.6) | 6.1 | (4.3;8.0) | 4.7 | (3.1;6.2) | 5.4 | (3.3;7.5) | 4.9 | (3.4;6.4) | 4.1 | (2.8;5.4) | 7.8 | (5.8;9.8) | 7.2* | (1.7 | ; | 13.1) |  |  |
| High | 3.1 | (1.8;4.3) | 3.7 | (2.1;5.3) | 3.5 | (2.1;4.9) | 4.0 | (2.6;5.4) | 3.5 | (2.1;4.9) | 4.5 | (3.1;5.9) | 4.2 | (2.7;5.8) | 4.3 | (2.7;5.8) | 5.7 | (3.7;7.7) | 6.0 | (4.1;7.9) | 6.8 * | (4.2 | ; | 9.4) |  |  |
| Values are presented as weighted % (95% confidence interval). Age-standardized prevalence was calculated using the 2005 Population Projections for Korea.  *The annual percent change (APC) is significantly different from 0. | | | | | | | | | | | | | | | | | | | | | | | | | | |
