# Supplementary material for: Obesity, hypertension, diabetes mellitus, and hypercholesterolemia in Korean adults before and during the COVID-19 pandemic: a special report of the 2020 Korea National Health and Nutrition Examination Survey
Source: Epidemiol Health. 2022 Apr 25;44:e2022041. doi: 10.4178/epih.e2022041 (PMC9133598; doi:10.4178/epih.e2022041)
Supplement: Supplementary Material 6 — Prevalence by income level in obesity, hypertension, diabetes mellitus, and hypercholesterolemia. [file epih-44-e2022041-suppl6.docx]

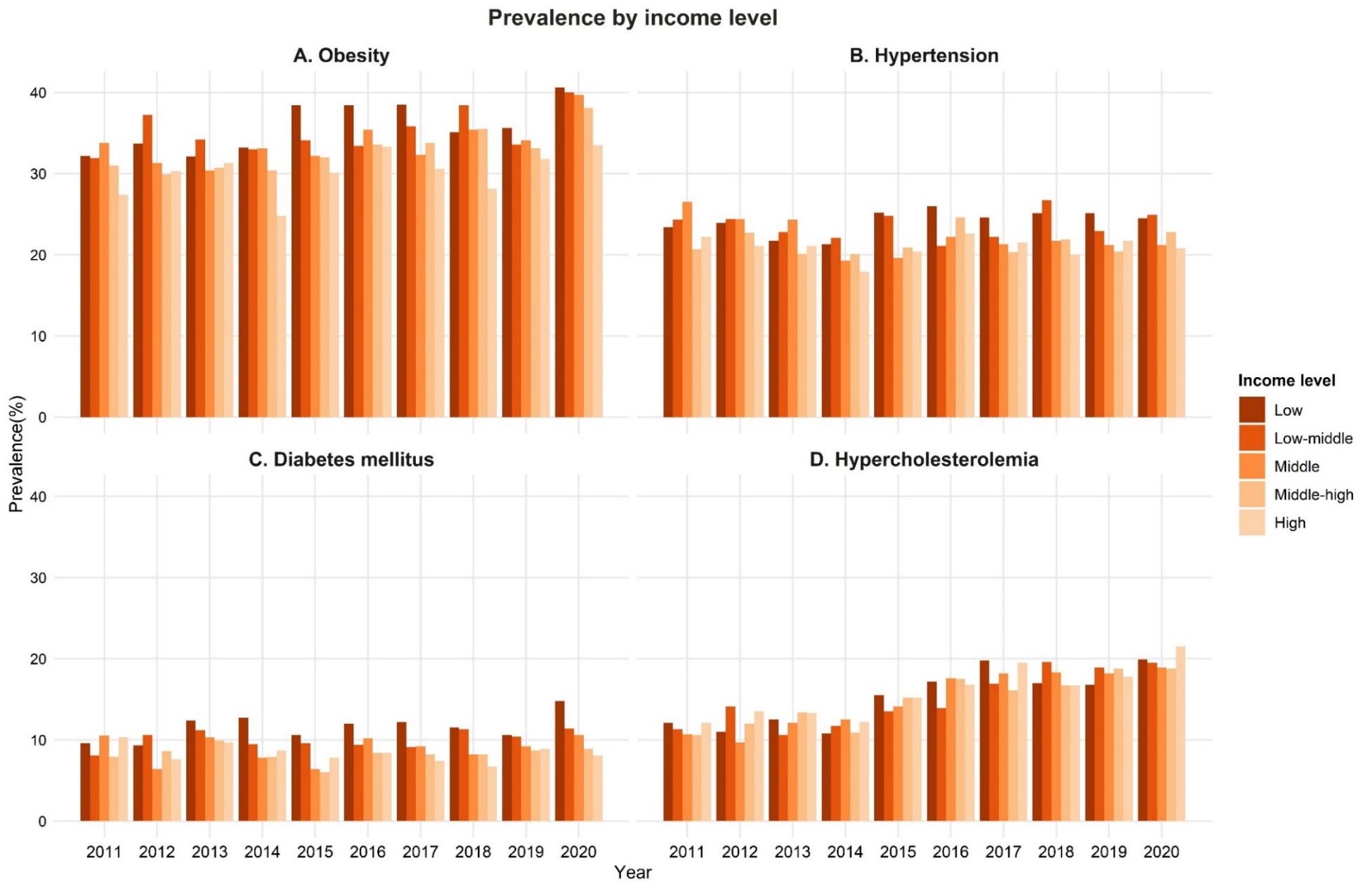
Supplementary Material 6. Prevalence by income level in obesity, hypertension, diabetes mellitus, and hypercholesterolemia.
